# Deciphering the Metabolic Pathway Difference Between *Saccharopolyspora pogona* and *Saccharopolyspora spinosa* by Comparative Proteomics and Metabonomics

**DOI:** 10.3389/fmicb.2020.00396

**Published:** 2020-03-18

**Authors:** Jie Rang, Haocheng He, Shuangqin Yuan, Jianli Tang, Zhudong Liu, Ziyuan Xia, Tahir Ali Khan, Shengbiao Hu, Ziquan Yu, Yibo Hu, Yunjun Sun, Weitao Huang, Xuezhi Ding, Liqiu Xia

**Affiliations:** Hunan Provincial Key Laboratory for Microbial Molecular Biology, State Key Laboratory of Development Biology of Freshwater Fish, College of Life Science, Hunan Normal University, Changsha, China

**Keywords:** *Saccharopolyspora pogona*, *Saccharopolyspora spinosa*, butenyl-spinosyn, spinosyn, comparative proteomic analysis, metabolic pathway, rhamnose synthetic genes, *metK*

## Abstract

Butenyl-spinosyn, a secondary metabolite produced by *Saccharopolyspora pogona*, exhibits strong insecticidal activity than spinosyn. However, the low synthesis capacity and unknown metabolic characteristics of butenyl-spinosyn in wild-type *S. pogona* limit its broad application and metabolic engineering. Here, we showed that *S. pogona* exhibited increased glucose consumption ability and growth rate compared with *S. spinosa*, but the production of butenyl-spinosyn was much lower than that of spinosyn. To further elucidate the metabolic mechanism of these different phenotypes, we performed a comparative proteomic and metabolomic study on *S. pogona* and *S. spinosa* to identify the change in the abundance levels of proteins and metabolites. We found that the abundance of most proteins and metabolites associated with glucose transport, fatty acid metabolism, tricarboxylic acid cycle, amino acid metabolism, energy metabolism, purine and pyrimidine metabolism, and target product biosynthesis in *S. pogona* was higher than that in *S. spinosa*. However, the overall abundance of proteins involved in butenyl-spinosyn biosynthesis was much lower than that of the high-abundance protein chaperonin GroEL, such as the enzymes related to rhamnose synthesis. We speculated that these protein and metabolite abundance changes may be directly responsible for the above phenotypic changes in *S. pogona* and *S. spinosa*, especially affecting butenyl-spinosyn biosynthesis. Further studies revealed that the over-expression of the rhamnose synthetic genes and methionine adenosyltransferase gene could effectively improve the production of butenyl-spinosyn by 2.69- and 3.03-fold, respectively, confirming the reliability of this conjecture. This work presents the first comparative proteomics and metabolomics study of *S. pogona* and *S. spinosa*, providing new insights into the novel links of phenotypic change and metabolic difference between two strains. The result will be valuable in designing strategies to promote the biosynthesis of butenyl-spinosyn by metabolic engineering.

## Introduction

Butenyl-spinosyn and spinosyn are secondary metabolites produced by *Saccharopolyspora pogona* (*S. pogona*) and *Saccharopolyspora spinosa* (*S. spinosa*), respectively. These two metabolites share similar chemical structure and belong to a new class of highly efficient and environment-friendly antibiotic insecticides that possess broad market potential (Dhakal et al., 2017; Lawler, 2017; White et al., 2017). These two analogs consist of a 21-carbon tetracyclic lactone containing the two deoxysugars forosamine and tri-O-methyl rhamnose, and their obvious difference is whether the C-21 position of the tetracyclic lactone system is attached to ethyl group (spinosyn) or butenyl group (butenyl-spinosyn) (Hahn et al., 2006; Lewer et al., 2009). The butenyl-spinosyn biosynthetic genes were initially cloned from *S. pogona* by using spinosyn biosynthetic genes as probes, and its coding sequences showed 94% identity to the DNA sequence of the spinosyn biosynthetic genes (Hahn et al., 2006). The only notable difference in the sequence comparisons was the presence of one additional polyketide synthase module in the butenyl-spinosyn gene sequence (*busA*) that was responsible for the two additional carbons in the C-21 tail of the spinosyn. This suggested that the spinosyn biosynthetic gene cluster might have been derived as an in-frame deletion from butenyl-spinosyn biosynthetic gene cluster. The biosynthetic pathways of butenyl-spinosyn and spinosyn are very similar (Jeon et al., 2017). These two metabolites are assembled by a polyketide pathway from acetate and propionate, forming a tetracyclic lactone, in which butenyl-spinosyn needs to consume an additional acetate. A neutral sugar (rhamnose) and an amino sugar (forosamine) are then coupled at the C9 and C17 site of the tetracyclic lactone, respectively. Rhamnose is subsequently methylated by three O-methyltransferases and eventually forms the final products butenyl-spinosyn and spinosyn (Huang et al., 2009). However, in the process of butenyl-spinosyn biosynthesis, amino sugars may be replaced by other sugar groups, such as amicetose, resulting in a wider variety of derivatives (Lewer et al., 2009).

Given the importance of butenyl-spinosyn and spinosyn, their insecticidal mechanism has been extensively studied in the past decades. These metabolites can kill susceptible insects by causing rapid excitation of the insect nervous system, probably through interaction and binding at nicotinacetylcholine and δ-amino butyric acid receptors (Millar and Denholm, 2007). Although butenyl-spinosyn and spinosyn share similar synthetic route and insecticidal mechanism, butenyl-spinosyn exhibits stronger insecticidal activity and broader insecticidal spectrum than spinosyn. For example, butenyl-spinosyn demonstrates good insecticidal activity against worldwide quarantine pest codling moth and the pest insect *Helicoverpa assulta* (Lewer et al., 2009; ValcÁrcel et al., 2015). Spinosyn has now been commercialized in products, such as Tracer, Success, SpinTor, Conserve, and Entrust (Blanc et al., 2004; Huang et al., 2009). The low biosynthetic ability of butenyl-spinosyn in wild-type *S. pogona* limits its commercial application. Hence, the production of butenyl-spinosyn must be improved.

At present, methods to enhance the butenyl-spinosyn and spinosyn production of strains have been extensively studied. The cyclic AMP receptor protein (*crp*) gene has been overexpressed in *S. spinosa*, improving spinosyns yield by 1.28-fold compared with that of the wild-type strain (Xu et al., 2014). A knock-out of *manB*, which codes for phosphomanmomutase in *S. spinosa*, increases spinosyn yield by 1.8-fold compared with the original strain (Liu et al., 2017). Other genes, such as *pepA* (Yang et al., 2016), *vhb* (Luo et al., 2012), and *metE* (Yang et al., 2015), can also affect spinosyn biosynthesis. Li et al. found that the over-expression of *pnp*, which encodes for polynucleotide phosphorylase in *S. pogona*, can affect strain growth and butenyl-spinosyn biosynthesis (Li et al., 2018). Although these reports demonstrate that some genes play important roles in the biosynthesis of target products, their production is not remarkably increased. Therefore, new target genes that directly affect the biosynthesis of butenyl-spinosyn and spinosyn must be urgently discovered.

Primary metabolic activities, such as central carbon metabolism, amino acid metabolism, energy metabolism, purine and pyrimidine metabolism play an important role in strain growth and secondary metabolite biosynthesis (Sun et al., 2017; Schniete et al., 2018). Wang et al. (2017a) discovered that central carbon metabolism influences cellulase production in *Bacillus licheniformis*. Sainz et al. (2017) reported that amino acids affect the growth of *Gluconobacter* and *Acetobacter*. Acetyl-CoA, malonyl-CoA, methylmalonyl-CoA, rhamnose, forosamine and S-adenosyl methionine are important precursors for butenyl-spinosyn biosynthesis and are also available from primary metabolic pathways, such as acetyl-CoA produced by glycolysis pathway and fatty acid degradation metabolism, S-adenosylmethionine produced by the cysteine and methionine metabolism, indicating a novel link between primary metabolism and butenyl-spinosyn biosynthesis. However, the key functional genes affecting the butenyl-spinosyn biosynthesis in primary metabolism pathway have not been fully studied.

In the current study, we aimed to explore the effect of metabolic differences on strain growth, glucose consumption, phosphate utilization, and antibiotic biosynthesis by comparing the phylogenetically coherent and functionally similar strains *S. pogona* and *S. spinosa* and to identify a series of key functional genes. We first analyzed the difference on strain growth, glucose consumption, phosphate utilization, and target product production between the two strains and found that *S. pogona* exhibited stronger glucose and phosphate consumption ability and growth rate but showed low target product biosynthetic ability compared with *S. spinosa*. Proteomics and metabolomic was then used to compare the differences in gene expression and metabolite abundance between the two strains under the same growth conditions. Through Gene Ontology (GO) and Kyoto Encyclopedia of Genes and Gnomes (KEGG) database analysis, we focused on the changes in the abundance of proteins and metabolites associated with primary metabolic pathways and the synthetic pathway of target products. We found at least 48 differential abundant proteins that may greatly affect strain growth, glucose and phosphate utilization, and butenyl-spinosyn biosynthesis. Using these datasets, we over-expressed the rhamnose synthetic genes and methionine adenosyltransferase gene in *S. pogona* and greatly increased the production of the target product. The results observed in this paper not only aided in elucidating how the metabolic differences affect phenotypic changes in *S. pogona* and *S. spinosa* but also provided key target genes for promoting butenyl-spinosyn biosynthesis.

## Materials and Methods

### Bacterial Strains and Growth Conditions

The bacterial strains used in this study are listed in Table 1. All *Escherichia coli* strains were grown at 37°C in Luria-Bertani broth (Jira et al., 2018). The spores of *S. pogona* and *S. spinosa* and their derivatives were activated in complete synthetic medium (per liter: 10 g of glucose, 45 g of trypticase soy broth, 9 g of yeast extract, and 2.2 g of MgSO_4_⋅7H_2_O). The bacteria were cultured at 30°C for 48 h, and a 2.5 mL aliquot of the seed culture was transferred into a 300 mL baffled flask containing 50 mL of synthetic fermentation medium (SFM; per liter: 1 g of KNO_3_, 0.01 g of FeSO_4_⋅7H_2_O, 0.5 g of K_2_HPO_4_⋅3H_2_O, 0.5 g of MgSO_4_⋅7H_2_O, 20 g of glucose, 4 g of yeast extract, 4 g of tryptone; pH of 7.2) and incubated at 30°C (Yang et al., 2014). If necessary, 50 μg/mL apramycin (Apra) was added into the medium.

**TABLE 1:** Strains and plasmids used in this study.

**Strains and plasmids**	**Relevant description**	**Source**
**Strains**		
*Escherichia coli* DH5α	Host for general cloning	This lab
*S. spinosa*	Spinosad producing strain	This lab
*S. pogona*	Butenyl-spinosad producing strain	This lab
SPOG-RM	*S. pogona* harboring pJNRM	This study
SPOG-ME	*S. pogona* harboringpKCcas9-*metK*	This study
**Plasmids**		
pMD18-T Vector	*E. coli* cloning vector, containing Amp^R^ and *LacZ*	
pMDGK	pMD18-T containing *gdh+kre*	This study
pMDGT	pMD18-T containing *gtt*	This study
pMDE	pMD18-T containing *epi*	This study
pMDRM	pMD18-T containing *gdh+kre*, *gtt* and *epi*	This study
pMDME	pMD18-T containing *metK*	This study
pJN100	*E. coli-Streptomyces* shuttle expression vector	This lab
pKCcas9dO	*E. coli-Streptomyces* shuttle expression vector	This lab
pJNRM	pJN100 containing *gdh+kre*, *gtt* and *epi*	This study
pKCcas9-*metK*	pKCcas9dO containing *metK*	This study

### Cultivation Profile Analysis of *S. pogona* and *S. spinosa*

To monitor the growth profile of *S. pogona* and *S. spinosa* in the SFM, the optical density of the culture at 600 nm (OD_600_) was analyzed to determine its cell concentration during fermentation. The cells were collected every 12 h for strain growth curve measurement by UV scanning. Supernatants were collected every 12 h during fermentation to determine the glucose concentration by using glucose assay kit (A031, Huilishengwu) until the glucose was used up. Supernatants were collected every 48 h during fermentation to determine the phosphate concentration by phosphomolybdenum blue spectrophotometry (Sun and Wang, 2014). To compare the production difference of butenyl-spinosyn and spinosyn produced by *S. pogona* and *S. spinosa*, respectively, we collected the cells at the 10th day in SFM for quantification and identification using HPLC and LC-MS/MS; sample processing and testing were conducted as described previously with slight modification (Lewer et al., 2009; Tang et al., 2011). Final culture was mixed with equal volume of acetone for 2 h, followed by centrifugation at 10,000 g for 10 min. After filtration through 0.22 μm Millipore filters, the supernatants (10 μL) were analyzed by high-performance liquid chromatography (Agilent 1290, United States) using a C18 reverse-phase column (AQ12S05-1546WT). For spinosyn, the column was developed at a flow rate of 0.5 mL/min by mobile phase methanol/acetonitrile/2% aqueous ammonium acetate (v/v/v = 45:45:10), each sample lasted 10 min with the isocratic elution (100% B) (12). While for butenyl-spinosyn, it was washed at a flow rate of 0.5 mL/min by gradient elution with mobile phase A (10 mM ammonium acetate) and mobile phase B (methanol/acetonitrile 1:1), each sample lasted 20 min with the following gradient: 60% B (2.5 min hold) ramped to a final mobile phase concentration of 100% B for 10.5 min (3 min hold). The two analogs were monitored at a wavelength of 250 nm. Then, the chromatographic peaks of the target products were verified by Thermo LTQ XL ion trap mass spectrometry in the positive spray ionization mode (Thermo Scientific, United States). The 15 μL samples (Chromatographic peak collected) were loaded onto a C18 column (LAGV-25005-102130, Thermo Scientific, United States) respectively and gradient eluted with the elution buffer at 300 μL/min. The elution buffer composed of solvent A (water, 0.1% formic acid, v/v) and solvent B (methanol, 0.1% formic acid, v/v). For spinosyn A and D, each chromatographic separation lasted 13 min with the following gradient: 50% B ramped to a final mobile phase concentration of 80% B for 6 min (2 min hold). For butenyl-spinosyn, each chromatographic separation lasted 25 min with the following gradient: 50% B (4 min hold) ramped to a final mobile phase concentration of 100% B for 15 min (4 min hold). The mass analyzer was scanned over a mass range of 80–800 m/z. Data analysis and instrument operations were performed under Xcaliber software control. The *Helicoverpa armigera* bioassay was used to verify whether the collected chromatographic peak had insecticidal activity as described previously (Li et al., 2018). Fermentation experiments were conducted in triplicate.

### Sample Preparation for Proteome Analysis

*S. pogona* and *S. spinosa* cells were harvested (8,000 g, 10 min, 4°C) after 4 days of culture, washed four times by resuspending the cell pellet in 20 mL of fresh PBS (10 mM, pH 7.8, pre-chilled at 4°C), and quickly frozen in liquid nitrogen. Protein extraction was performed as described previously (Yang et al., 2014). Samples were prepared from three biological replicates. Each protein extract (300 μg) was reduced with 500 mM dithiothreitol (DTT) at room temperature for 60 min and then alkylated with 500 mM iodoacetamide at room temperature in the dark for 60 min. Excess iodoacetamide was quenched with 15 mM DTT for 15 min at room temperature. The sample solutions were then incubated overnight with trypsin at a trypsin/protein ratio of 1:50 (w/w) at 37°C. Tryptic peptides were desalted and concentrated on an Oasis HLB sample cartridge column (Waters Corporation, MA, United States). Subsequently, the samples were labeled with an iTraq reagent in accordance with the manufacturer’s protocol (ABSciex, Framingham, MA, United States) and then lyophilized for further 2D online LC-MS analysis.

### 2D-LC-MS/MS Analysis

2D chromatography was conducted on an Eksigent nanoLC-Ultra^TM^1D System (AB SCIEX, Concord, ON, United States). The lyophilized SCX fractions were re-dissolved in 2% acetonitrile and 0.1% formic acid and then loaded on a ChromXP C18 (3 μm, 120 Å) nanoLC trap column. Online trapping and the desalting were performed at 2 μL/min for 10 min with 100% solvent A. The solvents were composed of water/acetonitrile/formic acid (A, 98%/2%/0.1%; B, 2%/98%/0.1%). An elution gradient of 5–38% acetonitrile (0.1% formic acid) in 70 min gradient was then employed on an analytical column (75 μm × 15 cm C18, 3 μm, 120 Å, ChromXP Eksigent). LC-MS/MS analysis was performed using a TripleTOF 5600 System (SCIEX, Concord, ON, United States) fitted with a Nanospray III source. Data were acquired at an ion spray voltage of 2.4 kV, a curtain gas of 30 PSI, a nebulizer gas of 5 PSI, and an interface heater temperature of 150°C. MS was operated with TOF-MS scans. For information dependent acquisition (IDA), the survey scans were acquired in 250 ms, and up to 30 product ion scans (80 ms) were collected if the threshold of 260 counts was exceeded and with a + 2 to + 5 charge state. Rolling collision energy setting was applied to all precursor ions for collision-induced dissociation. Dynamic exclusion was set to 16 s.

### Protein Identification and Quantification

MS/MS data were analyzed for protein identification and quantification using the ProteinPilot software v.4.5 (Sciex Inc., United States). The utilized protein database was the protein sequence set of all *Saccharopolyspora* strains. False discovery rate (FDR) was estimated via a reverse database strategy, and only proteins below the threshold of 1% of FDR were further considered. The differential abundant proteins (DAPs) were defined in the iTraq experiment on the basis of the following criteria: unique peptides ≥ 1, *P*-value < 0.05, and fold change > 1.33 or < 0.75. Only spots present in all the replicate gels were considered for subsequent analysis. The DAPs were annotated using the GO database^1^, and proteins were classified based on functional annotations using the GO terms for cellular components, biological processes, and molecular functions. The metabolic pathway analysis of DAPs was conducted using the KEGG database^2^.

### Determination of Intracellular Metabolites by UHPLCQ-TOF/MS

For metabolomic analysis, samples were collected at 4 days and from three biological replicates. The sample treatment methods used were as described by Wentzel et al. with some modifications (Wentzel et al., 2012). 10 mL of culture broth were collected and centrifuged, the samples were transferred to centrifuge tubes (1.5 mL) and mixed with 400 μL of methanol/acetonitrile (1:1, v/v). The tubes were vortexed for 30 s, incubated for 10 min at −20°C, and then centrifuged at 14,000 g for 15 min at 4°C. The supernatants were collected and dried with nitrogen, and then, the lyophilized powder was stored at −80°C prior to analysis. Lyophilized samples were reconstituted by dissolving in 100 μL of solvent mixture containing water/acetonitrile (5:5, v/v). The samples were vortexed for 1 min and centrifuged at 14,000 g for 15 min at 4°C. The supernatants were subjected to UHPLCQ-TOF/MS analysis. In parallel with the preparation of the test samples, pooled quality control (QC) samples were prepared by mixing equal amounts (30 μL) of each sample. The QC samples were utilized to monitor the UHPLC-Q-TOF/MS response in real-time.

Metabolic profiling of samples was performed on an Agilent 1290 Infinity LC system (Agilent Technologies, Santa-Clara, CA, United States) coupled with an AB SCIEX Triple TOF 5600 System (AB SCIEX, Framingham, MA, United States). Chromatographic separation was conducted on ACQUITY HSS T3 1.8 μm (2.1 × 100 mm) columns for both positive and negative models. The column temperature was set at 25°C for operation. The mobile phases of 0.1% formic acid in water (A) and 0.1% formic acid in acetonitrile (B) were used in positive ionization mode, while 0.5 mM ammonium fluoride in water (C) and acetonitrile (D) were used in negative ionization mode. In the positive (negative) model, the elution gradient initially started with 1% B (D) for 1 min, linearly increased to 100% B (D) at 8 min, was maintained for 2 min, and then returned to 1% B (D) for approximately 2 min of equilibrium. The delivery flow rate was 300 μL/min, and a 2 μL aliquot of each sample was injected onto the column. TOF/MS was performed on both ionization modes. Electrospray ionization source conditions on triple TOF were set as follows: ion source gas 1, 40 psi; ion source gas 2, 60 psi; curtain gas, 30 psi; source temperature, 650°C; ion spray voltage floating, 5000 V(+) and −4500 V (−). Information-dependent acquisition, an artificial intelligence-based product ion scan mode, was used to detect and identify MS/MS spectra. The parameters were set as follows: declustering potential, 60 V (+) and −60 V (−); collision energy, 50 V (+) and −20 V (−); exclude isotopes within 4 Da, candidate ions to monitor per cycle: 10. The analysis process was conducted with the assistance of Applied Protein Technology Co., Ltd. (Shanghai, China).

### Metabolomic Data Analysis

The raw data generated by UPLC-Q-TOF/MS were converted into mzML format files using the Proteo Wizard MS converter tool and then subjected to data processing using XCMS online software^3^. The data were subsequently processed using XCMS software for peak alignment and data filtering. For peak picking, the following parameters were used:centWave m/z = 25 ppm, peak width = c(10,60), prefilter = c(10, 100). For peak grouping, bw = 5, mzwid = 0.025, minfrac = 0.5 were used. Compound identification of metabolites was performed by comparing of accuracy m/z value (<25 ppm), and MS/MS spectra with an in-house database established with available authentic standards. After normalized to total peak intensity, the processed data were uploaded into SIMCA-P (version 14.1, Umetrics, Umea, Sweden) for statistical analysis. Partial-least squares discrimination analysis (PLS-DA) was conducted as a supervised method to identify the important variables with discriminative power. PLS-DA models were validated based on the multiple correlation coefficient (R2) and cross-validated R2 (Q2) in cross-validation and permutation tests by applying 200 iterations (*P* > 0.001). The significance of the biomarkers was evaluated by calculating the variable importance in projection (VIP) score (>1) from the OPLS-DA model. For the univariate analysis, specific biomarkers were compared between *S. pogona* and *S. spinosa* by employing Student’s *T*-test. A *p*-value less than 0.05 indicated a significantly different metabolite.

### Over-Expression of the Rhamnose Synthetic Genes and Methionine Adenosyltransferase Gene in *S. pogona*

The gene fragments *gdh* + *kre*, *gtt*, and *epi* were amplified from the genomic DNA of *S. spinosa* by using primer pairs P1/P2, P3/P4, and P5/P6 (Supplementary Table 1), respectively. The PCR products of *gdh* + *kre*, *gtt*, and *epi* were ligated to pMD18-T to generate the plasmids pMDGK, pMDGT, and pMDE, respectively. To construct all of the four genes in one vector, we first cloned them into a pMD18-T clone vector by restriction enzyme digestion and ligation. Subsequently, the clone was subcloned into the *E. coli–Streptomyces* shuttle expression vector pJN100 to obtain pJNRM. The gene fragment *metK* was amplified from the genomic DNA of *S. pogona* by using primer pairs P7/P8 (Supplementary Table 1). The PCR product of *metK* was ligated to pMD18-T to generate the plasmid pMDME. Subsequently, the clone was subcloned into the *E. coli–Streptomyces* shuttle expression vector pKCcas9dO to obtain pKCcas9-*metK*.

The pJNRM and pKCcas9-*metK* vectors were then transferred into *S. pogona* through protoplast transformation by employing a previously published method (Ma et al., 2014), respectively. Apramycin (50 μg/mL) was used to select transformants. The transformants named SPOG-RM and SPOG-ME were confirmed by PCR using primers for the apramycin resistance gene (Supplementary Table 1).

### RNA Isolation and qRT-PCR

For RNA isolation for quantitative reverse transcription-PCR (qRT-PCR), all strains were cultivated in SFM at 30°C. Four-day cultures (1 mL) were collected, and the cells were harvested via centrifugation at 8,000 g for 5 min at 4°C. Total RNAs were separately isolated using TRIzol Reagent (Invitrogen). RNA concentration and purity were determined by measuring the ratio of OD_260_ nm to OD_280_ nm. cDNA synthesis was performed using a High-Capacity cDNA Archive kit (Fermentas) in accordance with manufacturer’s instructions. qRT-PCR amplification was performed using Power SYBRR Green PCR Master Mix (Applied Biosystems), as previously described (Yang et al., 2014). The primer sequences used in qRT-PCR were designed with Primer Premier 5.0 and are listed in Supplementary Table 1. The transcript generated from the 16S rRNA gene was used for normalization. Results are presented as the means of four replicate experiments.

### Statistical Analysis

All data in this study were stated as means ± Standard deviation (SD), and analysis by Student’s *t*-test, with ^∗^*p* < 0.05, ^∗∗^*p* < 0.01, ns, no significant.

## Results and Discussion

### Qualitative and Quantitative Analyses of Butenyl-Spinosyn and Spinosyn in SFM

**FIGURE 1:**
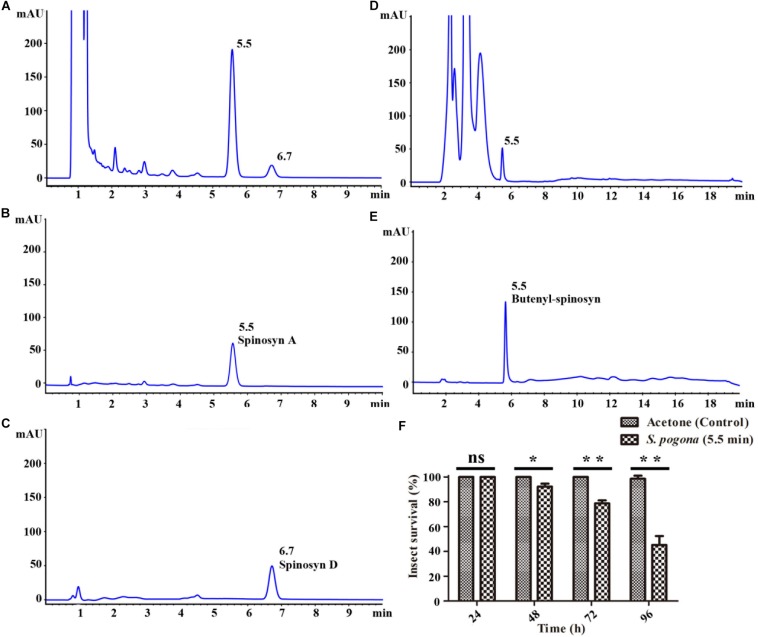
HPLC analysis of spinosyn and butenyl-spinosyn. **(A)** Fermentation broth of wild-type strain *S. spinosa*. **(B)** Standard spinosyn A. **(C)** Standard spinosyn D. **(D)** Fermentation broth of wild-type strain *S. pogona*. **(E)** Purified butenyl-spinosyn. **(F)** Analysis of insecticidal activity of *H. armigera* of the eluent collected at 5.5 min. *p < 0.05, **p < 0.01, ns, no significant.

Butenyl-spinosyn and spinosyn were extracted from the SFM of *S. pogona* and *S. spinosa* and then examined through HPLC and LC-MS/MS in accordance with the method described above (Lewer et al., 2009; Tang et al., 2011). The results of HPLC test showed that the fermentation broth of *S. spinosa* had obvious chromatographic peaks at 5.5 and 6.7 min respectively (Figure 1A), while the fermentation broth of *S. pogona* had obvious chromatographic peaks at 5.5 min (Figure 1D), and their characteristic absorption wavelength was 250 nm. For *S. spinosa*, all tested samples revealed two chromatographic peaks with the same retention times as those of the standard spinosyn A and spinosyn D (Figures 1B,C). The eluent collected were further confirmed by LC-MS/MS, showing a measured m/z of 732.6 and 747.0 (M + H)^+^ which were consistent with the molecular formula C_41_H_65_NO_10_ for spinosyn A and C_42_H_67_NO_10_ for spinosyn D, respectively (Supplementary Figures 1, 2). For *S. pogona*, bioassay result showed that the collected eluent corresponding to the chromatographic peak at 5.5 min had obvious insecticidal activity against the larvae of *Helicoverpa armigera* (Figures 1E,F). Then, the eluent collected was separated and purified several times to obtain relatively pure chromatographic peaks, and a substance with a m/z = 650 was identified through LC-MS analysis, and a rhamnose ion fragment with a m/z = 189 can also be identified by secondary mass spectrometry analysis (Supplementary Figure 3). Compared with the structures of butenyl-spinosyn derivatives reported by Paul et al. (2014), this substance was identified as the butenyl-spinosyn components spinosyn αc. Next, the chromatographic peak area was used as a basis for comparing the production differences of butenyl-spinosyn and spinosyn. The peak area of butenyl-spinosyn in the *S. pogona* was 472.8 ± 59.05 mAU*s, while the total peak area of spinosyn A and D in the *S. spinosa* was 2330.1 ± 87.75 mAU*s. This result indicates *S. pogona* exhibited lower synthesis ability for butenyl-spinosyn compared with that of *S. spinosa* for spinosyn, although butenyl-spinosyn exhibits a strong insecticidal effect (Li et al., 2018).

### Cultivation Profile Analysis of *S. pogona* and *S. spinosa*

**FIGURE 2:**
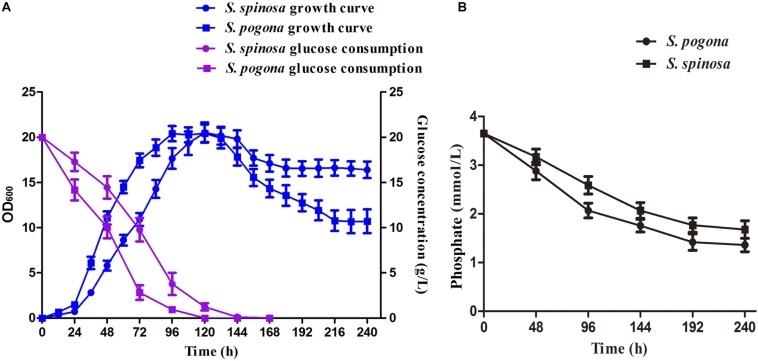
Comparison of growth characteristics between *S. pogona* and *S. spinosa*. **(A)** Growth kinetics and glucose consumption analysis. **(B)** Determination of phosphate in SFM.

Bacterial biomass and precursor supply are the main factors controlling secondary metabolite biosynthesis (Latha et al., 2017). We hypothesized that the strain growth rate and precursor supply ability of *S. pogona* and *S. spinosa* may show significant differences. Under most circumstances, glucose is the preferred carbon source for living organisms (Wu et al., 2016; Zeng et al., 2017). In the present study, we selected a glucose-rich culture medium and performed a comparative analysis of strain growth and glucose consumption in *S. pogona* and *S. spinosa* (Figure 2A). We first analyzed cell concentration by determining OD_600_ and found that *S. pogona* quickly entered the logarithmic phase at the duration of approximately 96 h. In contrast with the long logarithmic phase, a relatively short stationary phase (24 h), followed by rapid entry into the decline phase was shown by *S. pogona*. For *S. spinosa*, its growth rate was slower than that of *S. pogona* early in growth. However, its bacterial density decreased slowly during the recession, which the final bacterial density of *S. spinosa* was 1.65-fold that of *S. pogona* (Figure 2A). We continued to analyze glucose consumption in *S. pogona* and *S. spinosa* and found that the glucose utilization was in agreement with the growth tendency of these two strains, which was rapidly reduced during the logarithmic phase and was almost exhausted during the stationary phase. In *S. pogona*, however, glucose utilization was fast, and glucose was almost exhausted at 96 h, causing *S. pogona* to prematurely enter into the stationary and decline phases during the middle stage due to insufficient glucose (Figure 2A). In the soil-dwelling actinomycetes, the concentration of inorganic phosphate in the culture media is one of the most important nutritional factors affecting both growth and differentiation, and secondary metabolite biosynthesis (Antonio et al., 2010). We analyzed the phosphate consumption in *S. pogona* and *S. spinosa* and found that the phosphate concentration in the extracellular environment of the *S. pogona* was lower than that of the *S. spinosa* in early growth, which means that the utilization of phosphate by *S. pogona* is higher than *S. spinosa* (Figure 2B). The differences in strain growth, glucose and phosphate utilization indicated considerable metabolic differences between *S. pogona* and *S. spinosa*.

### Profiling of *S. pogona* and *S. spinosa* Proteome Under Glucose-Rich Conditions and Major Protein Expression Pattern Analysis

To further understand the causes of the phenotypic differences between *S. pogona* and *S. spinosa*, we examined the protein expression profiles in glucose-containing SFM. Samples for proteome profiling were obtained at stationary phases (4 days) to analyze the key factors affecting the phenotypic changes. Proteins isolated from three independent samples within the same phase were mixed and fragmented using trypsin. The resulting mass spectrum data were searched against the *Saccharopolyspora* proteome database. A total of 5,207 proteins were identified, 2,894 of which were identified in all of the samples, which were common proteins identified by *S. pogona* and *S. spinosa*, representing approximately 29.80 and 32.15% of the predicted proteome for *S. pogona* and *S. spinosa*, respectively (Data Set S1 and Supplementary Figure 4). A previous proteomics study on *S. spinosa* found 1,579 expressed proteins (17.54% of predicted proteome) (Yang et al., 2014), which was remarkably lower than the 2,894 expressed proteins (32.15% of predicted proteome) identified in this present study, indicating the feasibility of analytical method.

**FIGURE 3:**
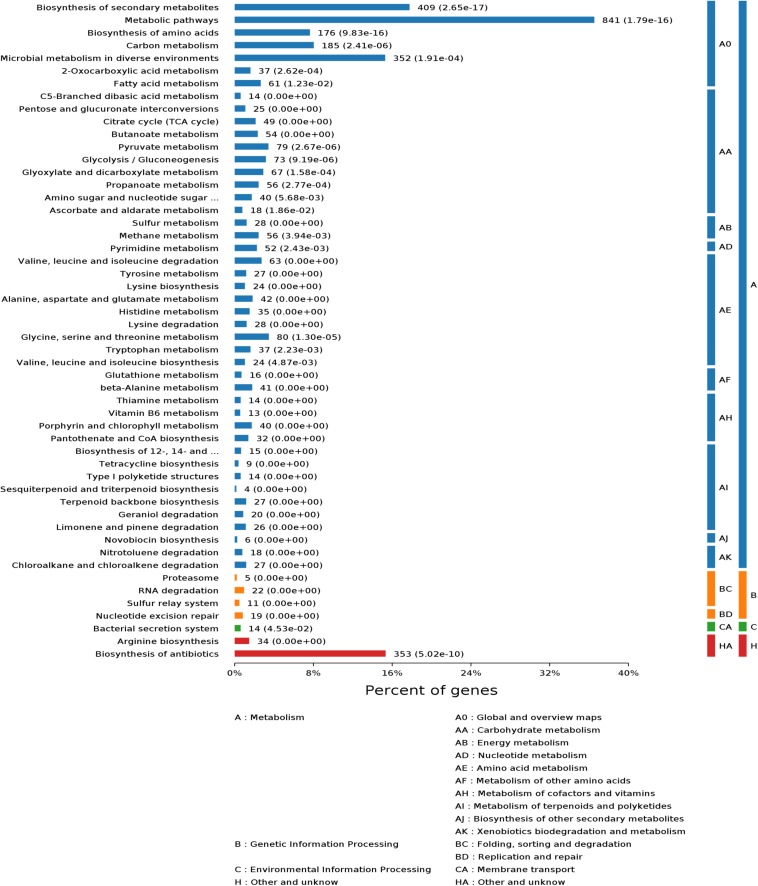
Kyoto encyclopedia of genes and genomes (KEGG) functional classification of the common proteins in *S. pogona* and *S. spinosa*. The number of genes associated with each pathway is indicated.

To evaluate the possible function of these proteins, we carried out GO and KEGG functional annotation analysis (Figure 3 and Supplementary Figure 5). The analysis results showed that these proteins were widely involved in metabolism, genetic information processing, and environmental information processing. To reveal the metabolic differences between *S. pogona* and *S. spinosa* from the biological system level, we estimated the abundance value of all proteins identified in this study, and the protein abundance scales are listed in Dataset S1. Some similarities and differences existed in the most highly expressed proteins between the two strains (Table 2). The most abundant protein in *S. pogona* and *S. spinosa* was the chaperonin GroEL, which assists in protein folding to ensure the success of important biological processes and respectively accounts for 4.35 and 6.41% of the species’ non-redundant proteome. In the present study, other proteins, including elongation factor Tu, aconitate hydratase AcnA, and F0F1 ATP synthase subunit alpha/beta, were also highly expressed in *S. pogona* and *S. spinosa*, indicating that these two strains exhibited strong metabolism and translation abilities. However, among the top 15 high-abundance proteins, five unique proteins were observed in each strain (Table 2). These proteins included type I glutamate-ammonia ligase, a hypothetical protein (WP_029535058.1), ATP-dependent Clp protease ATP-binding subunit, phosphopyruvate hydratase, and polynucleotide phosphorylase in *S. pogona* and cyclic nucleotide-binding domain-containing protein, methionine adenosyltransferase, type I glyceraldehyde-3-phosphate dehydrogenase, molecular chaperone DnaK, and NDMA-dependent alcohol dehydrogenase in *S. spinosa*. The differential expression of these high-abundance proteins likely affected the phenotypic changes between the two strains. For example, methionine adenosyltransferase (*metK*) can catalyze L-methionine conversion into S-adenosyl-L-methionine, which is an important source for butenyl-spinosyn and spinosyn methylation (Yang et al., 2015). However, its detection abundance of in *S. spinosa* was remarkably higher than that of *S. pogona* (2.41-fold), which may ultimately affect the biosynthesis of target products. These high-abundance proteins play a role in key metabolic pathways, such as glycolysis, tricarboxylic acid (TCA) cycle, oxidative phosphorylation, and RNA degradation, as well as basic cell function and maintenance.

**TABLE 2:** Most highly expressed proteins from *S. pogona* and *S. spinosa* under glucose-rich conditions.

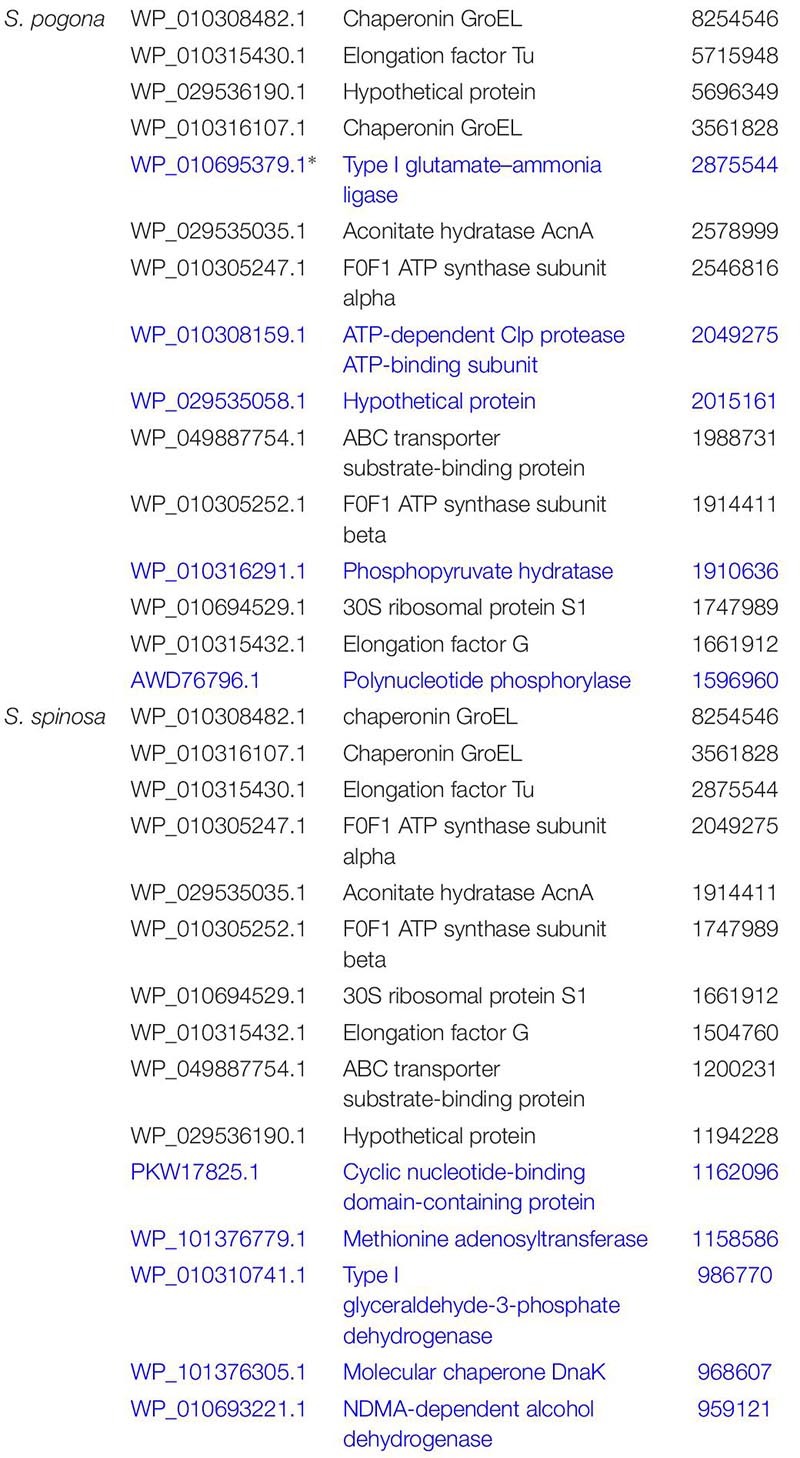

*The first 15 high-abundance proteins are specific to S. pogona and S. pinosa (blue mark).

### Metabolomic Analysis of the Key Metabolites and Pathways Associated With Strain Growth Development and Target Products Biosynthesis

**FIGURE 4:**
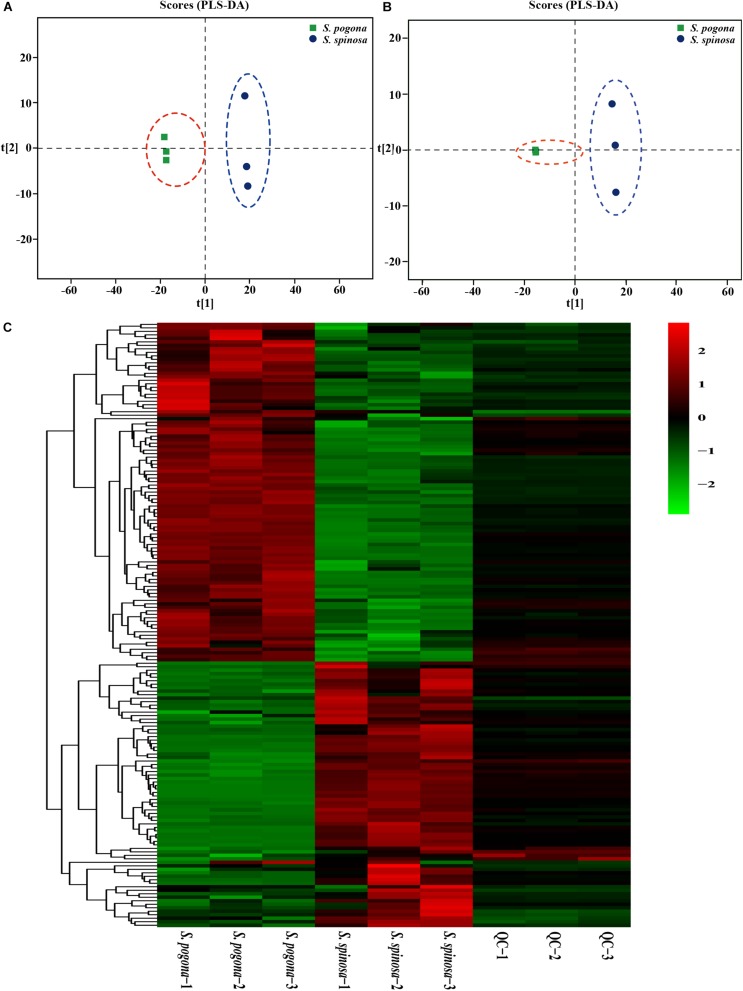
PLS-DA and heatmap analysis of intracellular metabolites from *S. pogona* and *S. spinosa*. **(A)** PLS-DA score graph of positive ion model. **(B)** PLS-DA score graph of negative ion model. **(C)** Heatmap analysis of significantly different metabolites in different samples.

In order to analyze whether the changes of metabolite abundance related to strain growth development and target products biosynthesis were consistent with the changes of proteins, detection of intracellular metabolome was carried out by UHPLCQ-TOF/MS (Fu et al., 2019). In this study, the samples at stationary phases (4 days) were also used for metabolomic profiling to analyze the correlation with proteomic data. As a result, 235 intracellular metabolites were identified and quantified by UHPLCQ-TOF/MS, including sugars, amino acids, organic acids, fatty acids, phospholipids and their derivatives (Data Set S2). Next, PLS-DA modeling was employed to evaluate the relationship between metabolite expression and sample difference (Sindt et al., 2018). The R2(X), R2(Y), and Q2 of PLS-DA model for positive and negative ion scanning were 0.779, 1, 0.994, and 0.771, 1, 0.993, respectively, highlighting the reliability of the PLS-DA analysis (Figures 4A,B). To further identify which metabolites were closely associated with the sample difference, the VIP was generated by OPLS-DA analysis. A higher VIP score suggested that the metabolites contributed more significantly to sample difference (Wang et al., 2017b). A total of 150 identified metabolites were selected (VIP score > 1, Fold change > 1.5 and < 0.67, and *p* < 0.05) for further investigation. Abundances of all the metabolites are shown in Figure 4C by the heat map. These metabolites were mainly involved in glycolysis, TCA cycle, fatty acid degradation metabolism, amino acid metabolism, purine and pyrimidine metabolism, and most of them were related to the synthesis of acetyl-CoA, rhamnose, forosamine and S-adenosyl-L-methionine. In addition, we found that the detection abundance of phospholipid molecules involved in cell membrane composition in *S. pogona* was mostly higher than that of the *S. spinosa*, such as phosphorylcholine (4.57), 1-oleoyl-sn-glycero-3-phosphocholine (6.77), glycerophosphocholine (3.42), phosphatidylethanolamine (1.83), etc. (Data Set S3). These detection results effectively reflected the difference in metabolic pathways between the *S. pogona* and *S. spinosa*.

### Central Carbon Metabolism

**FIGURE 5:**
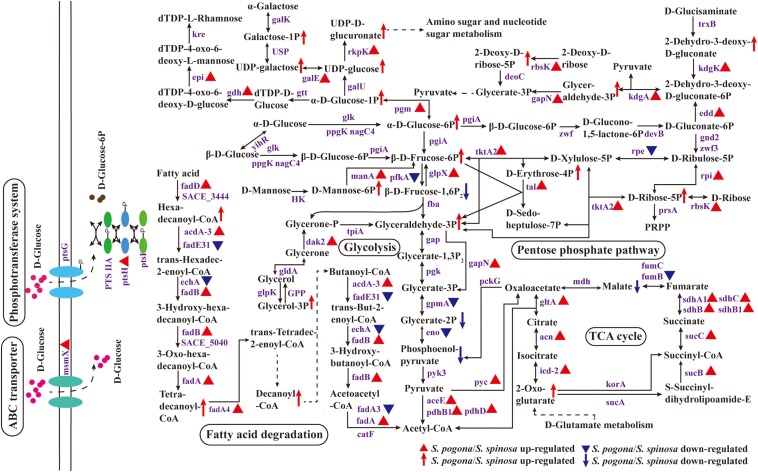
Protein and metabolite changes of the central carbon metabolic pathway in *S. pogona* and *S. spinosa*. The protein changes are from iTraq-labeled identification results. The proteins indicated in triangle pattern (red or blue) represent significant differences in *S. pogona*. The metabolite changes are from metabolomic analysis results. The metabolites indicated in arrow (red or blue) represent significant differences in *S. pogona*. TCA cycle, citrate cycle; PTS system, phosphotransferase system; P, phosphate; PRPP, 5-phospho-alpha-D-ribose 1-diphosphate.

Central carbon metabolism is one of the most basic cellular pathways that occur in all living organisms, and metabolite interconversion at the phosphoenolpyruvate–pyruvate–oxaloacetate node involves a set of reactions that connect the major pathways of carbon metabolism (Sauer and Eikmanns, 2005; Wittmann et al., 2012). These reactions are responsible for the distribution of carbon flux among catabolism, anabolism, and cellular energy supply (Chubukov et al., 2014). The current study presents an overview of the central carbon metabolic pathway and changes in the expression levels of proteins involved in key metabolism, as shown in Figure 5. Most proteins involved in central carbon metabolism could be identified from the shotgun proteomic data (Data Set S4). Cells convert extracellular glucose into the available phosphorylated glucose, glucose 6-phosphate, via two pathways. First is direct phosphorylation, in which glucose is transported into cells through the phosphotransferase system (PTS); second is the entry of glucose into cells via the ATP-binding cassette (ABC) transport system, followed by the phosphorylation of glucose by glucokinase (Zhou et al., 2015; Xia et al., 2017). In the present study, PTS enzyme I (*ptsI*), phosphocarrier protein Hpr (*ptsH*), PTS glucose transporter IIA/IIBC (PTS IIA/IIBC), multiple sugar transport system ATP-binding protein (*msmX*), and glucokinase (*ppgK*) were identified in *S. pogona* and *S. spinosa* during glucose fermentation. The abundance of HPr and multiple sugar transport system ATP-binding protein in *S. pogona* was higher than that of *S. spinosa*, indicating that both strains have developed ways of glucose transport by PTS and the ABC transport system. Furthermore, the ability of *S. pogona* to transport and phosphorylate glucose was stronger than that of *S. spinosa*, which was kept in line with the rapid glucose consumption rate and the improvement of cell growth. In addition, the expression abundance of the proteins related to the transport of fructose, mannose and other sugars in *S. pogona* was also higher than that of *S. spinosa*, indicating that the potential ability of *S. pogona* to transport other sugars was probably stronger than that of *S. spinosa* (Data Set S1).

Three common alternative routes for glucose catabolism occur in bacteria: the Embden-Meyerhof-Parnas (glycolysis) pathway, Entner-Doudoroff (ED) pathway, and pentose phosphate (PP) pathway (Nikel et al., 2015; Seol et al., 2016; Sánchez-Pascuala et al., 2017). The glycolysis and PP pathways are nearly ubiquitous in the bacterial kingdom (Romano and Conway, 1996). In the current study, *S. pogona* and *S. spinosa* could catabolize glucose into acetyl-CoA via the complete glycolysis and PP pathways, and all of the proteins involved in these two pathways could be detected. Throughout the glycolysis process, the abundance of 6-phosphofructokinase (*pfkA*) in *S. pogona* was lower than that of *S. spinosa*; this enzyme is involved in the conversion of fructose-6-phosphate to fructose-1,6-bisphosphate and is a rate-limiting enzyme for glycolysis (Suo et al., 2018). The abundance of fructose-1,6-bisphosphatase (*glpX*) was higher than that of *S. spinosa*; this enzyme is involved in the conversion of fructose-1,6-bisphosphate to fructose-6-phosphate and reduce substrate metabolism flow to glycolysis (Gutka et al., 2017). Meanwhile, fructose-6-phosphate, as shown in the metabolomic analysis, it had a higher abundance in *S. pogona*, and the fructose-1,6-bisphosphate had a lower abundance in *S. pogona*, which was consistent with the abundance variation of 6-phosphofructokinase and fructose-1,6- bisphosphatase in *S. pogona*. Two other enzymes in glycolysis, namely, phosphoglycerate mutase (*gpmA*) and enolase (*eno*) (Nikel et al., 2015), were also observed, and their abundance in *S. pogona* was lower than that in *S. spinosa*. Moreover, their catalytic products, such as glycerate-2-phosphate and phosphoenolpyruvate, have a low abundance in *S. pogona*. The data indicate that the ability of *S. pogona* to utilize glucose by glycolysis on the 4th day was weaker than that of *S. spinosa*, which is probably caused by the low glucose concentration in the extracellular environment of *S. pogona*. Even so, the expression abundances of enzymes involved in the conversion of pyruvate to acetyl-CoA or oxaloacetate in *S. pogona*, such as pyruvate dehydrogenase (*aceE*, *pdhB1* and *pdhD*) and pyruvate carboxylase (*pyc*), was higher than that of *S. spinosa*, indicating that *S. pogona* has a strong ability to metabolize pyruvate. The PP pathway is the second important pathway for glucose metabolism, and its initial substrate is glucose 6-phosphate. In fast-growing cells, the PP pathway plays crucial roles in anabolic reactions, converting glucose 6-phosphate into glyceraldehyde 3-phosphate and ribose 5-phosphate or producing NADPH for the synthesis of acetyl-CoA, nucleotides, and other metabolites (Seol et al., 2016). The pathway can be divided into oxidation and non-oxidation stages. In oxidation stage, glucose 6-phosphate undergoes two-step dehydrogenation reaction to form ribulose 5-phosphate, CO_2_ and two molecules of NADPH, of which glucose-6-phosphate dehydrogenase (*zwf*) and 6-phosphogluconate dehydrogenase (*zwf3*) are the rate-limiting enzymes of this stage (Matsushika et al., 2012). However, the abundance of these proteins did not remarkably change in our comparative proteomic data, suggesting that these two strains showed the same ability to metabolize glucose through the oxidation stage of PP pathway. In non-oxidation stage, it is mainly involved in the mutual conversion of different carbon number compounds, in which transketolase (*tktA2*) and transaldolase (*tal*) play important roles in the above reactions (Tittmann, 2014). Surprisingly, the expression abundance of these two enzymes in *S. pogona* was significantly higher than that of *S. spinosa*. Moreover, some of the intermediate metabolites that occur in the non-oxidation phase have a higher abundance in *S. pogona*, such as erythrose-4-phosphate, ribose-5-phosphate and glyceraldehyde-3-phosphate, which indicated that the ability of different carbon number compounds mutual conversion through the non-oxidation stage of PP pathway in *S. pogona* was higher than that of *S. spinosa*. Unexpectedly, we also detected 6-phosphogluconate dehydratase (*edd*) and 2-dehydro-3-deoxy-D-gluconate aldolase (*kdgA*) in the proteomic data of *S. pogona* and *S. spinosa*, which were characteristic enzymes of the ED pathway that takes only four steps to convert glucose to pyruvate (Flamholz et al., 2013). Moreover, the abundance of 6-phosphogluconate dehydratase and 2-dehydro-3-deoxy-D-gluconate aldolase in *S. pogona* was higher than that of *S. spinosa*, indicating the strong ability of *S. pogona* to catabolize glucose through the ED pathway. Therefore, comparative proteomic analysis revealed three glucose metabolism modes in both *S. pogona* and *S. spinosa*, demonstrating their strong basal metabolic capacity. However, the ability of the two strains in glycolysis, PP pathway and ED pathway were remarkably different in a selected period, causing their specific phenotypic changes. For example, the growth rate of *S. pogona* was stronger than that of the *S. spinosa* in early growth.

Ribokinase (*rbsK*) was also identified in our proteomic data. Ribokinase is one of the principal enzymes in carbohydrate metabolism, catalyzing D-ribose and 2-deoxy-D-ribose to produce ribose-5-phosphate and 2-deoxy-D-ribose-5-phosphate, respectively, eventually generating glyceraldehyde-3-phosphate into glycolysis; this pathway may be used as an alternative route in central carbon metabolism (Paul et al., 2014; Zhou et al., 2019). We found that the abundance of ribokinase in *S. pogona* was higher than that of *S. spinosa*. Meanwhile, its catalytic products, such as ribose-5-phosphate and 2-deoxy-D-ribose-5-phosphate, have a higher abundance in *S. pogona*. The results suggest that the extracellular glucose deficiency of *S. pogona* improves the utilization of cells to intracellular D-ribose and 2-deoxy-D-ribose due to the absence of a “glucose effect” (Alba et al., 2016). The ribose-5-phosphate and 2-deoxy-D-ribose-5-phosphate generated can be used to produce glyceraldehyde 3-phosphate and NTP/dNTP via the PP pathway and via purine and pyrimidine metabolism to compensate for the shortage of glucose to complete normal metabolism.

In addition to catabolism through glycolysis and PP pathway, glucose-6-phosphate can also be converted into glucose-1-phosphate for anabolism, including polyketide sugar unit biosynthesis, N-glycan biosynthesis and peptidoglycan biosynthesis (Patel et al., 2019). In this study, the abundance of phosphoglucomutase (*pgm*) in *S. pogona* was higher than that of *S. spinosa*; this enzyme is involved in the conversion of glucose-6-phosphate to glucose-1-phosphate, and its catalyzed product, glucose 1-phosphate, also has a high abundance in *S. pogona*. Figure 5 showed that glucose-1-phosphate can synthesize dTDP-L-rhamnose successively with the participation of glucose-1-phosphate thymidylyltransferase (*gtt*), dTDP-glucose-4,6-dehydratase (*gdh*), dTDP-4-keto-6-deoxy-D-glucose epimerase (*epi*) and dTDP-4-dehydrorhamnose reductase (*kre*), and can also synthesize UDP-D-glucuronate successively with the participation of UTP-glucose-1-phosphate uridylyltransferase (*galU*) and UDP-N-acetyl-D-glucosamine dehydrogenase (*rkpK*), in which dTDP-L-rhamnose is an important precursor of target products and cell wall synthesis, while UDP-D-glucuronate is an important substrate for amino sugar and nucleotide sugar metabolism. In the above biological processes, the expression abundance of dTDP-glucose-4,6-dehydratase, dTDP-4-keto-6-deoxy-D-glucose epimerase and UDP-N-acetyl-D-glucosamine dehydrogenase in *S. pogona* was higher than that in *S. spinosa*. In addition, UDP-glucose and UDP-D-glucuronate also have a high detection abundance in *S. pogona*, indicating that *S. pogona* has a strong ability to use glucose for anabolism during the analysis period. We also found that enzymes and intermediate metabolites associated with the metabolism of mannose, galactose, and glycerol 3-phosphate had high abundance in *S. pogona*, such as mannose-6-phosphate isomerase (*manA*), UDP-glucose 4-epimerase (*galE*), dihydroxyacetone kinase (*dak2*), D-mannose 6-phosphate, galactose 1-phosphate, UDP-galactose and glycerol 3-phosphate, which effectively reflected the difference on metabolic capacity between the two strains.

Fatty acid degradation metabolism and TCA cycle are important metabolic pathways for the synthesis of acetyl-CoA and other intermediate precursors, respectively (Edmunds et al., 2014; Vuoristo et al., 2017; Richie et al., 2018). Bacteria use several mechanisms for activating exogenous fatty acids, one of the most important of which is the conversion of exogenous fatty acids to acyl-CoA, which is then broken down by β-oxidation to produce acetyl-CoA (Kallscheuer et al., 2017). The proteomic data in the present study showed that the abundance of acyl-CoA synthetase (*fadD*), acyl-CoA dehydrogenase (*acdA-3*), enoyl-CoA hydratase (*fadB*), and acetyl-CoA acyltransferase (*fadA*, *fadA4*) in *S. pogona* were higher than that in *S. spinosa*. Meanwhile, hexadecanoyl-CoA, tetradecanoyl-CoA and decanoyl-CoA had a higher abundance in *S. pogona*, which was consistent with the abundance variation of acyl-CoA synthetase and acetyl-CoA acyltransferase in *S. pogona*. Since acyl-CoA synthetase is the core enzyme for fatty acid degradation metabolism, this result suggests the strong ability of *S. pogona* to metabolize exogenous fatty acids by β-oxidation. The acetyl-CoA produced from fatty acid β-oxidation and pyruvate metabolism will enter the TCA cycle to produce energy and several metabolic precursors for cell growth and the synthesis of other metabolites. Citrate synthase (*gltA*), isocitrate dehydrogenase (*icd-2*), and 2-oxoglutarate dehydrogenase (*sucB*) are the rate-limiting enzymes of TCA cycle and were detected in our proteomic data. The abundance of these three rate-limiting enzymes in *S. pogona* was higher than that in *S. spinosa*. The same finding was observed for other enzymes involved in the TCA cycle, such as aconitate hydratase (*acn*), succinyl-CoA synthetase (*sucC*), and succinate dehydrogenase (*sdhA1*, *sdhB*, *sdhB1*, and *sdhC*). In addition, we also found that 2-oxoglutarate has a high abundance in *S. pogona*. This result suggests the strong ability of *S. pogona* to produce energy and metabolic precursors by using acetyl-CoA, although the concentration of fumarate hydratase (*fumB*) and its catalytic product malate is low. *S. pogona* showed stronger metabolic capacity than *S. spinosa* in fatty acid metabolism and TCA cycle because it rapidly absorbs and utilizes nutrients in the environment early in growth. It suggests that the metabolic flux flowing through the TCA cycle in *S. pogona* is increased compared with the *S. spinosa*, which may cause the acetyl-CoA produced by the glycolysis, fatty acid degradation and other metabolic pathways to flow more into the TCA cycle, and ultimately limits the biosynthesis of butenyl-spinosyn. These results can be used to explain why *S. pogona* exhibited a rapid growth rate but low butenyl-spinosyn production.

### Amino Acid Metabolism

**FIGURE 6:**
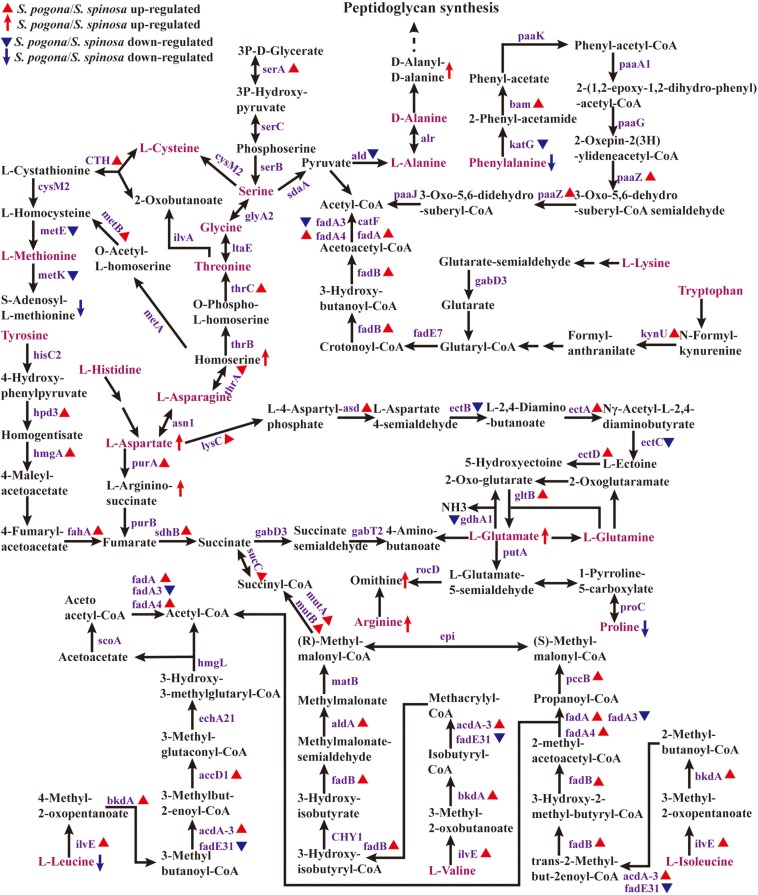
Protein and metabolite changes of the amino acids metabolism in *S. pogona* and *S. spinosa*. The protein changes are from iTraq-labeled identification results. The proteins identified are indicated in purple. The proteins indicated in triangle pattern (red or blue) represent significant differences in *S. pogona*. The metabolite changes are from metabolomic analysis results. The metabolites indicated in arrow (red or blue) represent significant differences in *S. pogona*. The multistep reaction is indicated in consecutive arrows. P, phosphate.

Amino acid metabolism plays an important role in cell growth and secondary metabolite biosynthesis (Ljungdahl and Daignan-Fornier, 2012; Sainz et al., 2017). To further understand how amino acid metabolism functions in target product biosynthesis, we analyzed the expression of enzymes for amino acid metabolism and the detection abundance of intermediate metabolites (Figure 6, Data Set S2 and S4). Figure 6 shows that leucine, valine, isoleucine, methionine, serine, alanine, phenylalanine, lysine, and tryptophan can be directly converted into important precursors of target product biosynthesis, such as acetyl-CoA, methylmalonyl-CoA, and S-adenosyl-L-methionine. Histidine, tyrosine, asparagine, aspartate, cysteine, glycine, threonine, glutamate, and glutamine indirectly synthesize these important precursors. However, arginine and proline cannot be converted to these precursors. This means that amino acid metabolism is also an important source of precursors for the biosynthesis of target products. We found that the amino acid metabolism ability of *S. pogona* overall was stronger than that of *S. spinosa*, indicating that *S. pogona* demonstrated a stronger synthetic ability to convert amino acids into acetyl-CoA and other key precursors compared with *S. spinosa.* For example, the abundance of enzymes (*ilvE*, *bkdA*, *acdA-3*, *fadB*, *fadA*, *fadA4*, *pccB*, and *aldA*) involved in the leucine to acetyl-CoA, valine to (R)-methylmalonyl-CoA, and isoleucine to (S)-methylmalonyl-CoA pathways in *S. pogona* was remarkably higher than that in *S. spinosa* (Schertl et al., 2017). Meanwhile, leucine (0.36) and isoleucine (0.71) were detected at lower concentrations in *S. pogona* than in *S. spinosa*. This result indicates that *S. pogona* can rapidly convert the above amino acids into acetyl-CoA and methylmalonyl-CoA. However, we found that the abundance of homocysteine methyltransferase (*metE*) and methionine adenosyltransferase (*metK*), two key enzymes involved in the S-adenosine methionine synthesis (Yang et al., 2015), was remarkably lower in *S. pogona* than in *S. spinosa*, with a detection abundance of only 52 and 54%, respectively. This result may lead to insufficient S-adenosine methionine synthesis in *S. pogona*. As shown in the metabolome analysis, the detection abundance of S-adenosine methionine in *S. pogona* was also lower than that of *S. spinosa*, therefore, insufficient supply of S-adenosine methionine was one of the important factors resulting in the low butenyl-spinosyn yield. Further experiments are required to verify the role of S-adenosine methionine on butenyl-spinosyn biosynthesis.

### Energy Metabolism

**FIGURE 7:**
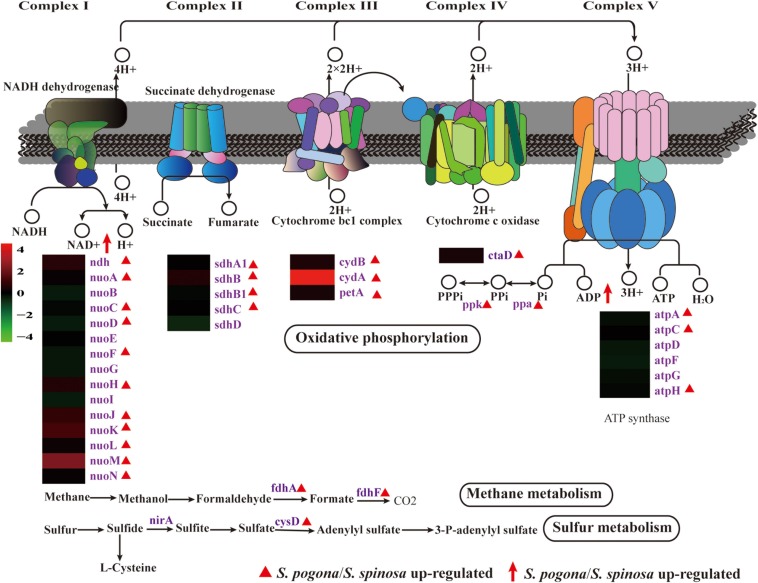
Protein and metabolite changes of energy metabolism in *S. pogona* and *S. spinosa*. The protein changes are from iTraq-labeled identification results. The proteins identified are indicated in purple. The proteins indicated in triangle pattern (red) represent significant differences in *S. pogona*. The metabolite changes are from metabolomic analysis results. The metabolites indicated in arrow (red) represent significant differences in *S. pogona*.

Most of the intermediate precursors and reducing power generated by central carbon and amino acid metabolism follow the oxidative phosphorylation pathway for ATP synthesis (Daniel, 2019). We detected the complete oxidative phosphorylation pathway (complex I–V) at the protein level and the complex I-, III-, and IV-generated proton gradient using the appropriate substrate (Data Set S2 and Figure 7). The proton gradient generated can be used to create ATP via complex V (Daniel, 2019). Based on the comparative proteomic analysis, the abundance of most enzymes identified in *S. pogona*, such as NADH dehydrogenase (*nouA*/*C*/*E*/*H*/*J*/*K*/*L*/*M*/*N, ndh*), succinate dehydrogenase (*sdhA1*/*B*/*B1*/*C*), polyphosphate kinase (*ppk*), inorganic pyrophosphatase (*ppa*), and ATP synthase (*atpA*/*C*/*H*), was higher than that in *S. spinosa* and showed an increased energy production capacity. NAD + /NADH ratio and total NAD(H) play important roles for whole-cell biochemical redox transformations. As shown in the previously described central carbon metabolism process, four reactions that generate reducing power (NADH and FADH_2_) occur in the TCA cycle: isocitrate to 2-oxo-glutarate (NADH), 2-oxo-glutarate to succinyl-CoA (NADH), succinate to fumarate (FADH_2_), and malate to oxaloacetate (NADH) (Vuoristo et al., 2017). The abundance of enzymes (*icd-2*, *sucB*, and *sdhA1*/*B/B1*/*C*) involved in the conversion of isocitrate to 2-oxo-glutarate, 2-oxo-glutarate to succinyl-CoA, and succinate to fumarate in *S. pogona* was remarkably higher than that in *S. spinosa*, implying the strong ability of *S. pogona* to produce reducing power. We hypothesized that the increased NADH level induces the elevated expression of oxidative phosphorylation-related enzymes in *S. pogona*, which was consistent with the quantitative proteomic analysis.

Unexpectedly, methane and sulfur metabolism are the other energy metabolism mechanisms found in our proteomic data based on KEGG pathway analysis (Figure 7) (Hakobyan et al., 2018; Qian et al., 2019). The enzymes formaldehyde dehydrogenase (*fdhA*), formate dehydrogenase (*fdhF*), and sulfate adenylyltransferase (*cysD*), which are involved in methane metabolism and sulfur metabolism, were identified, indicating that in addition to using organic carbon to generate energy, utilizing inorganic carbon (methane) and inorganic sulfur to produce energy in *S. pogona* and *S. spinosa* is highly possible.

### Nucleotide Metabolism

**FIGURE 8:**
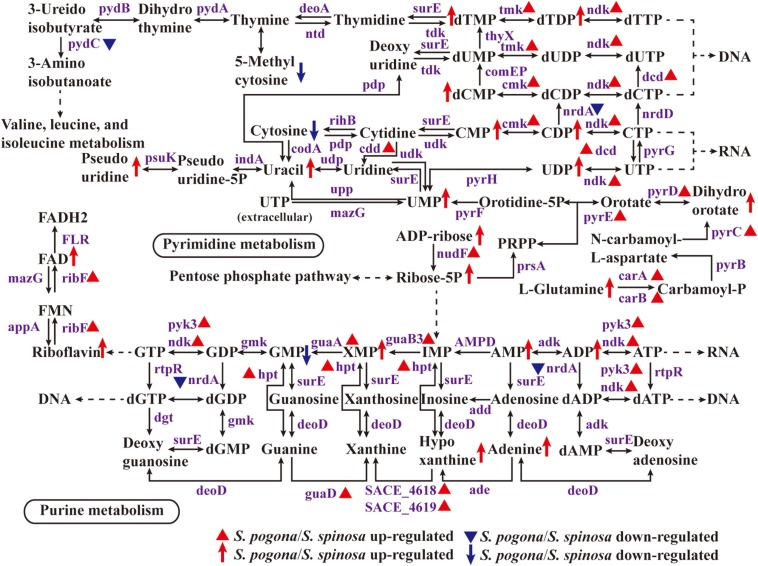
Protein and metabolite changes of nucleotide metabolism in *S. pogona* and *S. spinosa*. The protein changes are from iTraq-labeled identification results. The proteins identified are indicated in purple. The proteins indicated in triangle pattern (red or blue) represent significant differences in *S. pogona*. The metabolite changes are from metabolomic analysis results. The metabolites indicated in arrow (red or blue) represent significant differences in *S. pogona*.

Nucleotide metabolism plays an important role in the synthesis of genetic materials, energy supply, metabolic regulation, as a messenger molecule and the formation of coenzymes, including purine nucleotide metabolism and pyrimidine nucleotide metabolism (Kalia et al., 2013; Jenal et al., 2017). The synthesis of nucleotides in an organism can be through two pathways, one is *de novo* synthesis (primary) and the other is remediation synthesis (Wang, 2016). Figure 8 showed the gradual synthesis of nucleotides based on ribose 5-phosphate. In this study, it was found that most of the enzymes involved in nucleotide metabolism and their catalytic products had high abundance in *S. pogona*. IMP dehydrogenase (*guaB3*) catalyzes the redox reaction between inosine monophosphate (IMP) and xanthosine 5′-phosphate (XMP), carbamoyl-phosphate synthase (*carA*/*carB*) catalyzes the synthesis of carbamoyl-phosphate, which belongs to the rate-limiting enzyme of purine and pyrimidine nucleotide metabolism respectively (Rubio et al., 1998; Pimkin and Markham, 2009). The two enzymes as well as the relevant metabolites, such as xanthosine 5′-phosphate, L-glutamine and dihydroorotate, were all present at higher abundance in *S. pogona*, indicating that *S. pogona* has strong nucleotide metabolism ability. Moreover, in the previous analysis of the PP pathway, it was determined that enzymes involved in the synthesis of ribose 5-phosphate, such as ribose 5-phosphate isomerase (*rpi*), ribokinase and transketolase, had a higher expression abundance in *S. pogona*, making its ribose 5-phosphate abundance much higher than that of *S. spinosa*, so as to meet the needs of its vigorous nucleotide metabolism.

### Differential Analysis in the Expression of Proteins Directly Involved in Butenyl-Spinosyn Biosynthesis

In addition to metabolic pathways associated with precursor synthesis, the differential expression of the enzyme involved in the butenyl-spinosyn biosynthesis was also analyzed in this study (Table 3). In our shotgun proteomic data, 12 gene expression products directly related to the biosynthesis of butenyl-spinosyn were detected. These products were mainly involved in intramolecular C–C bond formation (*busJ*, *L*), rhamnose attachment and methylation (*busG*, *H*, *I*, *K*), forosamine biosynthesis (*busP*, *S*), and rhamnose biosynthesis (*gtt*, *gdh*, *epi*, and *kre*) (Huang et al., 2009). However, other proteins related to the biosynthesis of the butenyl-spinosyn could not be detected because of their low abundance. The expression abundance of these 12 proteins was much lower than that of certain high-abundance proteins, such as the molecular chaperone GroEL. For example, the expression abundance of BusI and BusP, the proteins with the highest and lowest abundance, were only the 1 and 0.05% that of GroEL, respectively. This result is a possible reason for the low butenyl-spinosyn synthesis rate in wild-type *S. pogona*.

**TABLE 3:** Proteins directly associated with butenyl-spinosyn or spinosyn biosynthesis identified by iTraq-labeled proteomic analysis.

**Accessions**	**Names**	**Abundance (*S. spinosa*)**	**Abundance (*S. pogona*)**	**Foldchanges**	***P-*value**
AAY88926.1	BusI/SpnI	36693	101102	2.76	0.04
AAY88927.1	BusJ/SpnJ	13308	28940	2.17	0.01
AAY88929.1	BusL/SpnL	12869	38104	2.96	0.01
AAY88925.1	BusH/SpnH	9213	20652	2.24	0.04
AAY88928.1	BusK/SpnK	5675	11732	2.07	0.04
AAY88924.1	BusG/SpnG	4490	10329	2.30	0.01
AAY88933.1	BusP/SpnP	3685	3918	1.06	0.80
AAY88936.1	BusS/SpnS	2321	3776	1.63	0.00
WP_010315535.1	glucose-1-phosphate thymidylyltransferase (Gtt)	28434	25312	0.89	0.06
WP_010315630.1	dTDP-glucose 4,6-dehydratase (Gdh)	6658	12518	1.88	0.00
WP_010304825.1	dTDP-4-keto-6-deoxy-D-glucose epimerase (Epi)	1960	5446	2.78	0.03
WP_029536157.1	dTDP-4-dehydrorhamnose reductase (Kre)	7733	7921	1.02	0.88

*Foldchanges: The comparison of protein abundance between S. pogona and S. spinosa (S. pogona/S. spinosa).*

### Effect of the Over-Expression of the Rhamnose Synthetic Genes and Methionine Adenosyltransferase Gene on the Butenyl-Spinosyn Biosynthesis

**FIGURE 9:**
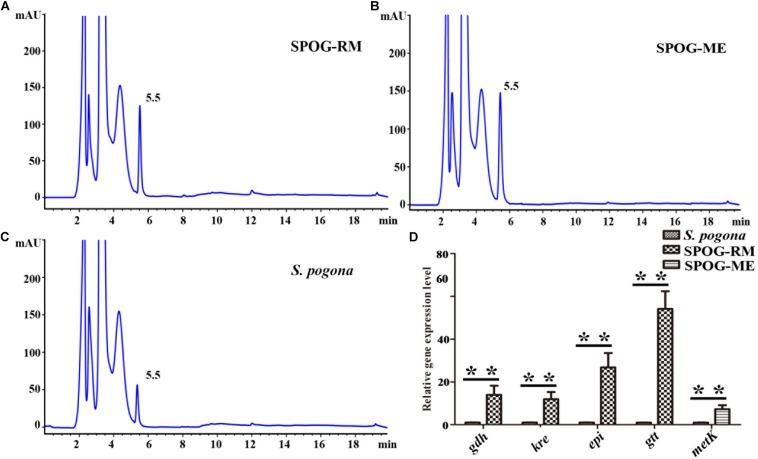
The over-expression of rhamnose synthetic genes and *metK* promote butenyl-spinosyn biosynthesis. **(A)** Fermentation broth of mutant SPOG-RM. **(B)** Fermentation broth of mutant SPOG-ME. **(C)** Fermentation broth of *S. pogona*. **(D)** Transcript analyses of rhamnose biosynthetic genes and *metK*. The cells of the different strains were cultured in SFM and incubated at 30°C for 4 days. Total RNA were then isolated and used for qRT-PCR assays. The control strain was the wild-type *S. pogona*. The ratio for the control strain was arbitrarily set to “1”. The 16s RNA served as the normalization control. Error bars were calculated from four independent determinations of mRNA abundance in each sample. The ** represents the *P*-value less than 0.01.

Rhamnose and S-adenosyl methionine are the precursors of butenyl-spinosyn biosynthesis. Meanwhile, the former is also participated in cell wall synthesis, and the latter is a direct donor of methyl groups. However, the proteomic results in the present study showed that the expression abundance of rhamnose synthetic genes (*gtt*, *gdh*, *epi*, and *kre*) and *metK* involved in the biosynthesis of rhamnose and S-adenosyl methionine was low compared to other high-abundance proteins (Table 3). Therefore, the lack of rhamnose and S-adenosyl methionine supply is likely to limit the butenyl-spinosyn biosynthesis. To confirm the conjecture, we over-expressed the entire rhamnose synthetic genes and *metK* in *S. pogona*, respectively, and then evaluated the resulting mutants SPOG-RM and SPOG-ME for discernable phenotypes (Supplementary Figures 6–8). The butenyl-spinosyn was also extracted from the mutants and then examined through HPLC in accordance with the method described above. The total peak area of butenyl-spinosyn reached 1220.7 ± 181.68 mAU*s in SPOG-RM and 1432.4 ± 164.24 mAU*s in SPOG-ME, and their production were enhanced by 2.69- and 3.03-fold compared with the original strain, respectively, which was consistent with our speculation (Figures 9A–C). We also analyzed the expression levels of rhamnose synthetic genes in SPOG-RM and *metK* in SPOG-ME. The qRT-PCR results showed that the transcript levels of *gdh*, *kre*, *epi*, and *gtt* in SPOG-RM were 14. 0-, 11. 9-, 26. 8-, and 54.1-fold higher than those in *S. pogona*, respectively, and the transcript level of *metK* in SPOG-ME was 7.3-fold higher than that in *S. pogona* (Figure 9D). The finding was also in agreement with the high fermentation observed in mutants. On the basis of metabolic pathway analysis and engineering modification, we believe that the insufficient supply of the synthetic units rhamnose and S-adenosine methionine is an important factor limiting butenyl-spinosyn biosynthesis.

## Conclusion

This study was the first to conduct a comparative analysis of *S. pogona* and *S. spinosa*. The results may contribute to our understanding of the biosynthetic mechanism of butenyl-spinosyn and spinosyn. Strain OD_600_ value, glucose consumption, phosphate utilization, and target product yield analysis indicated that *S. pogona* exhibited faster growth rate and glucose and phosphate utilization rate but lower target product biosynthetic ability compared with *S. spinosa*. Comparative proteomic and metabolomic analysis revealed the difference on central carbon metabolism, amino acid metabolism, energy metabolism, purine and pyrimidine metabolism, and the biosynthetic gene cluster expression of the target products between *S. pogona* and *S. spinosa*. These results suggest that *S. pogona* demonstrates high-efficiency glucose transport ability via the PTS and ABC transporter system, allowing its faster glucose utilization rate compared with that of *S. spinosa*. *S. pogona* showed strong ability for fatty acid metabolism, TCA cycle, amino acid metabolism, energy metabolism, purine and pyrimidine metabolism, allowing its vigorous basic metabolism to promote its rapid growth. However, this metabolic feature also led to insufficient precursors and energy supply of *S. pogona* during the butenyl-spinosyn biosynthesis, which not only caused its premature entry to the stationary and decline growth periods but also reduced its butenyl-spinosyn biosynthetic ability. The ability of *S. pogona* to participate in the synthesis of precursors, such as rhamnose and S-adenosyl methionine, was weaker than that of *S. spinosa*, thus affecting the precursor supply of butenyl-spinosyn biosynthesis. Proteomic data also showed that the overall expression level of the butenyl-spinosyn synthetic gene cluster was very low, which may greatly affect biosynthetic ability. The over-expression experiment of rhamnose synthetic genes and *metK* confirmed the reliability of this conjecture. We propose that the metabolic pathways associated with the supplies of rhamnose and S-adenosyl methionine, which were selected based on the results of quantitative proteomic and metabolomic data, require further research on their regulatory mechanism on butenyl-spinosyn biosynthesis. Studies on the genetic modification of their corresponding genes are useful to understand the key nodes and regulatory networks of primary metabolism and butenyl-spinosyn and spinosyn biosynthesis.

## Data Availability Statement

All datasets generated for this study are included in the article/Supplementary Material.

## Author Contributions

LX conceived the project. LX and JR generated the concepts and designed the research. JR, HH, SY, JT, and SH performed *S. pogona* and *S. spinosa* iTraq-based quantitative proteomic sample preparation and bioinformatics analysis. JR, HH, ZL, and ZX conducted mutants construction. JR, XD, HH, and ZY employed butenyl-spinosyn and spinosyn HPLC and MS analysis. HH, SH, TK, YH, and WH performed mutant strain physiological and biochemical assay. JR and LX wrote the manuscript. All authors discussed the results and approved the final manuscript.

## Conflict of Interest

The authors declare that the research was conducted in the absence of any commercial or financial relationships that could be construed as a potential conflict of interest.

## Footnotes

1 http://www.geneontology.org/

2 http://www.genome.jp/kegg/

3 https://xcmsonline.scripps.edu/landing_page.php?pgcontent=mainPage
