# Supplementary material for: Deciphering the Metabolic Pathway Difference Between Saccharopolyspora pogona and Saccharopolyspora spinosa by Comparative Proteomics and Metabonomics
Source: Front Microbiol. 2020 Mar 18;11:396. doi: 10.3389/fmicb.2020.00396 (PMC7093602; doi:10.3389/fmicb.2020.00396)
Supplement: Supplementary file 1 [file Data_Sheet_1.doc]

Supplementary Material

Table S1 Sequences of the primers used in this study.

| Name | Sequences |
| --- | --- |
| P1 | TATACCATGGAGGATTGGCGCGATGCGGATTCTGG |
| P2 | AATTCTTAAGGCTACTTGCGCCCTCCGGGTGACTG |
| P3 | TATAGCATGCTCAATAGAAATCGGACGAGCTCGGGTCAG |
| P4 | TATACTTAAGGAGGTATCCGGATGAAGGGGATCGTGCTG |
| P5 | TATACTCGAGGGAGGATTTAGGCCATGCAGGTACGTCGACTTG |
| P6 | TATAAAGCTTATATAAGCCGGGCCGCTTGCGGTGACCGTTCAG |
| P7 | TAATACTAGTGTGAGTGAGATCAACCGCA |
| P8 | GTCAAGCTTCGCACTGCATTACTCCTTCA |
| Apr-S | GTCCAATACGAATGGCGAAAAGC |
| Apr-A | ATAACATTCTTCGCATCCCGCC |
| snpA-left1 | CTATCGGCGGTTGCCATCATC |
| gtt-down2 | CGGCGCACCGGACTACGACAC |
| epi-in2 | TGTGGTTGGTCTGCGCGACTC |
| gdh-in2 | GAGCAACTGCCGAACGTAGTG |
| epi-down2 | TGGCCGAAGCCGGGCCGCTTG |
| 16s-S | GGGATAAGCCTTGGAAACGG |
| 16s-A | ATTCCCCACTGCTGCCTCC |
| gtt-rt-S | GACATGCCGTTGTTCCAG |
| gtt-rt-A | ACCGTCGTCGTCCACCT |
| gdh-rt-S | AGGTGCTGCCGCTGTTC |
| gdh-rt-A | TTCCTCGACGATCTTGCTG |
| epi-rt- S | CACGGGTGCATACGAGTTCA |
| epi-rt-A | CCGAGACGCTGTGGTTGGTT |
| kre-rt- S | CGGCAAGAACTTCCTGAAAACG |
| kre-rt-A | CCGAGACGCTGTGGTTGGT |
| metK-rt-S | GCTGACCAAGGTTCGCAAGG |
| metK-rt-A | TCTTCGAGGTCGATGTCGGC |

Note: Restriction enzyme sites were underlined.


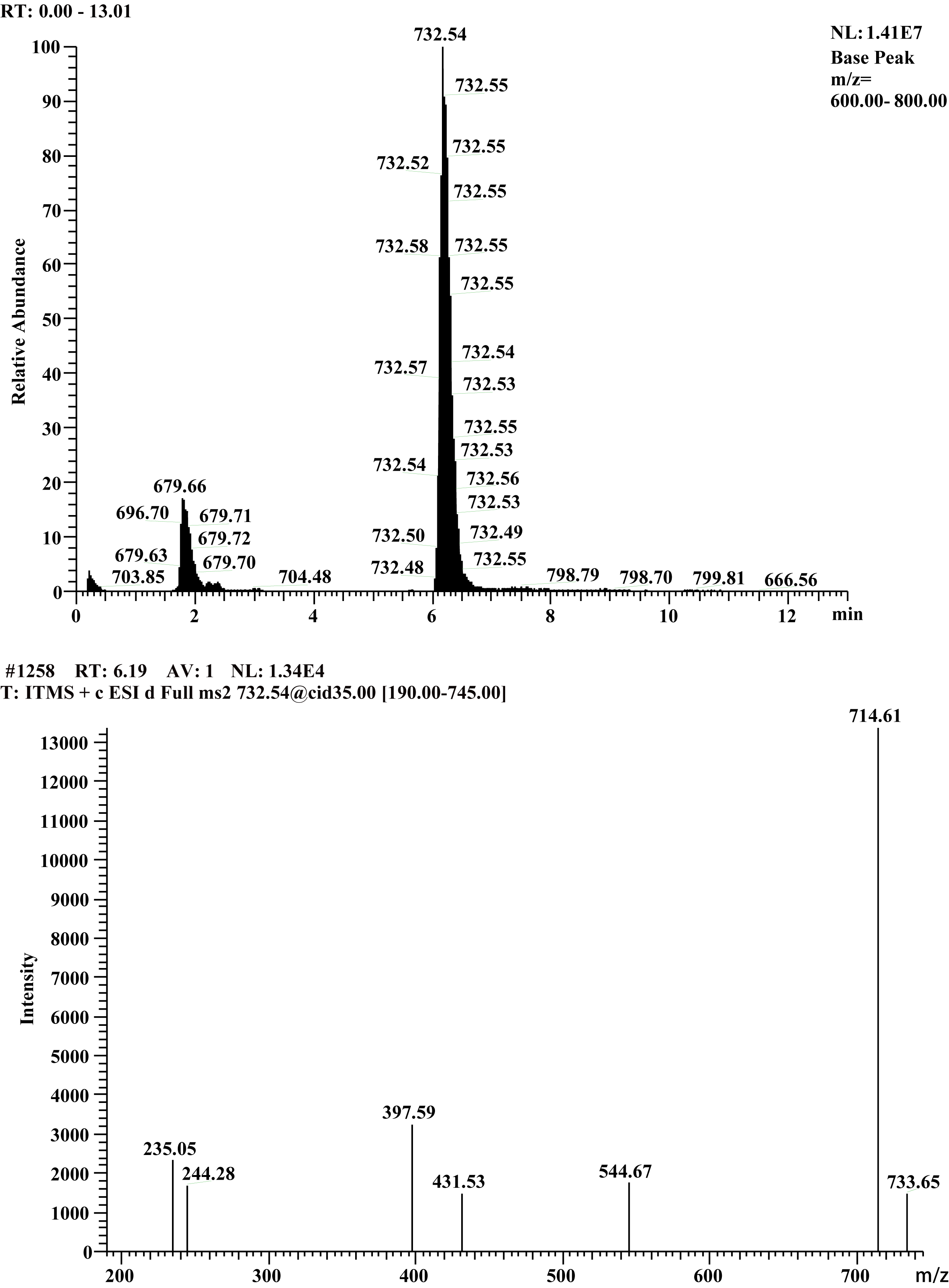


Supplementary Figure 1 | Mass spectrum identification of spinosyn A. (Up) Identification via MS. (Down) Identification via MS/MS.


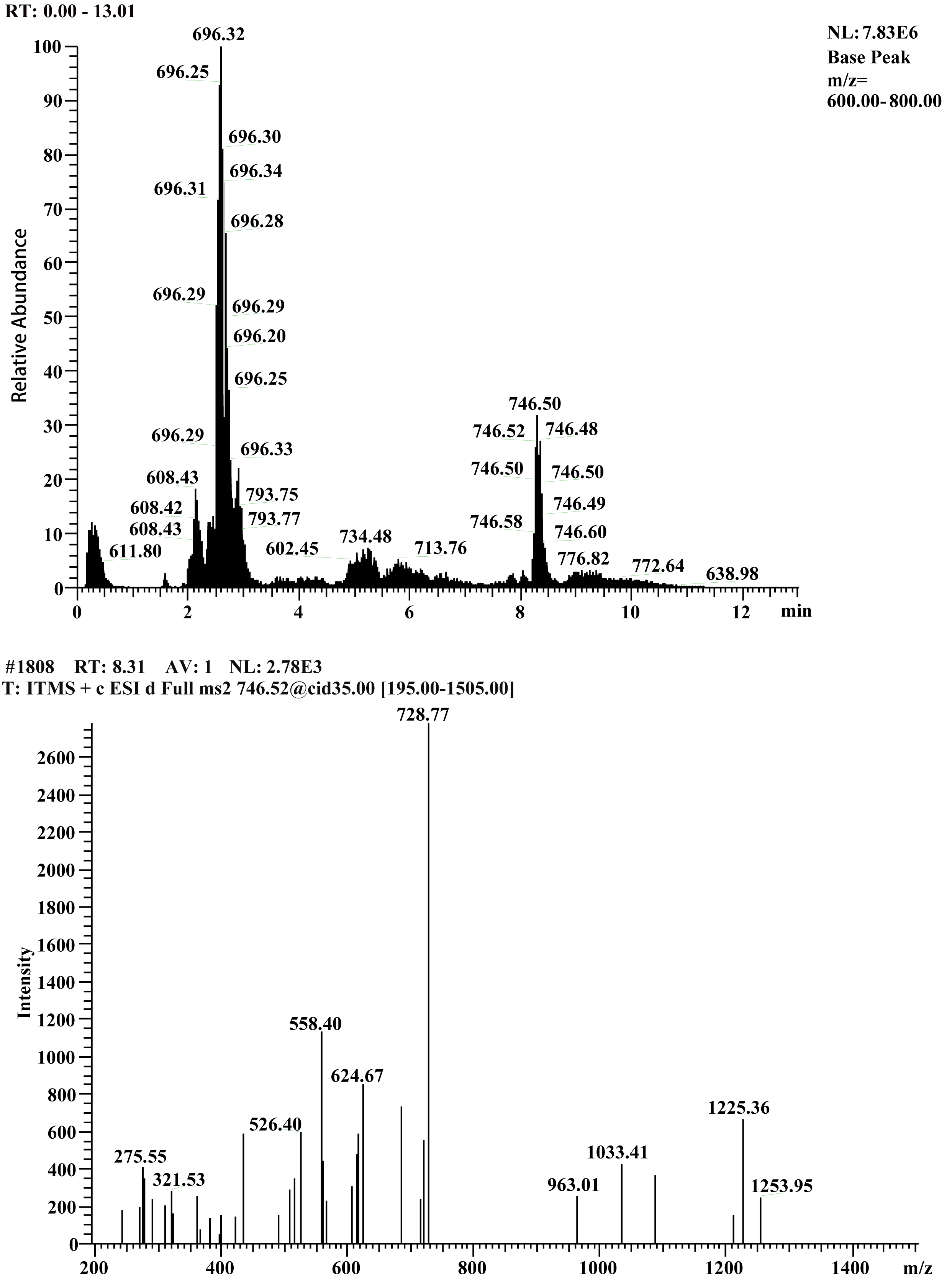


Supplementary Figure 2 | Mass spectrum identification of spinosyn D. (Up) Identification via MS. (Down) Identification via MS/MS.


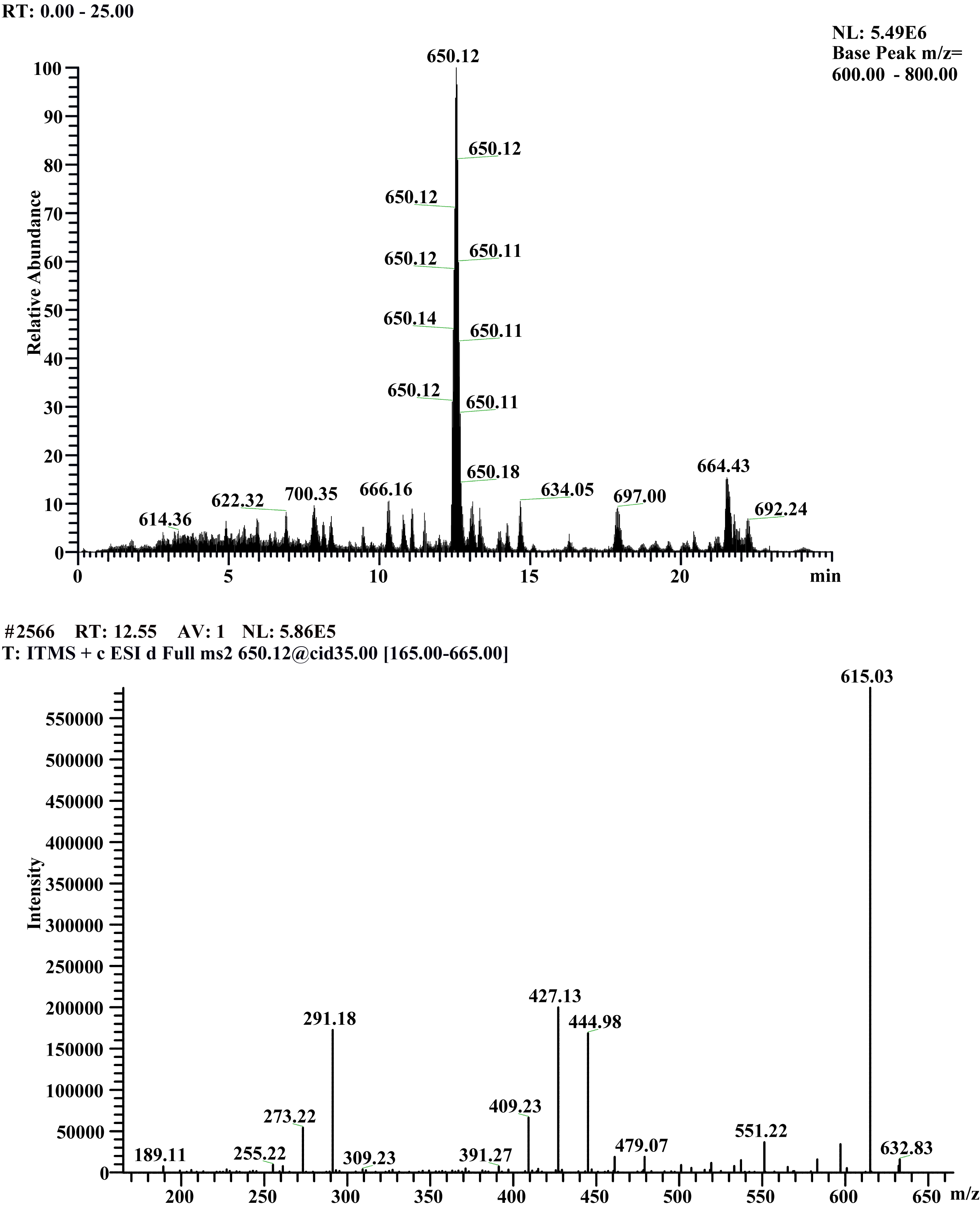


Supplementary Figure 3 | Mass spectrum identification of butenyl-spinosyn. (Up) Identification via MS. (Down) Identification via MS/MS.





Supplementary Figure 4 | Venn diagram of proteins identified at each sampling point for iTraq-labeled shotgun proteomic data. The number of proteins is shown in each area.





Supplementary Figure 5 | Functional classification of the common proteins identified in *S. pogona* and *S. spinosa* in terms of protein biological processes (A), molecular functions (B), and cell component (C).


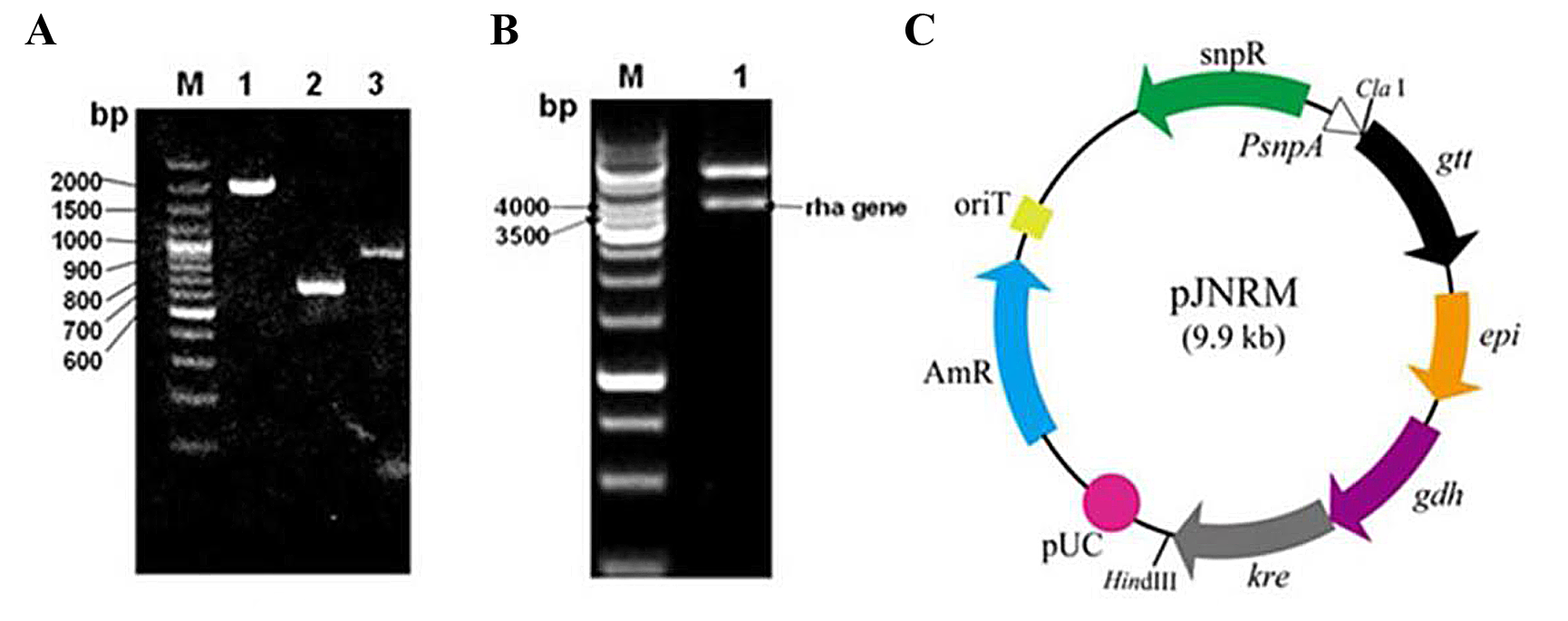


Supplementary Figure 6 | Construction of pJNRM rhamnose expression vector.

1. Agarose gel electrophoresis analysis of rhamnose genes (M, DNA marker. Lane 1, *gdh* + *kre*. Lane 2, *epi*. Lane 3, *gtt*). (B) Restriction enzyme analysis of pJNRM vector. (M, DNA marker. Lane 1, *Cla*I/*Hin*dIII double digestion). (C) The genetic map of pJNRM plasmid.


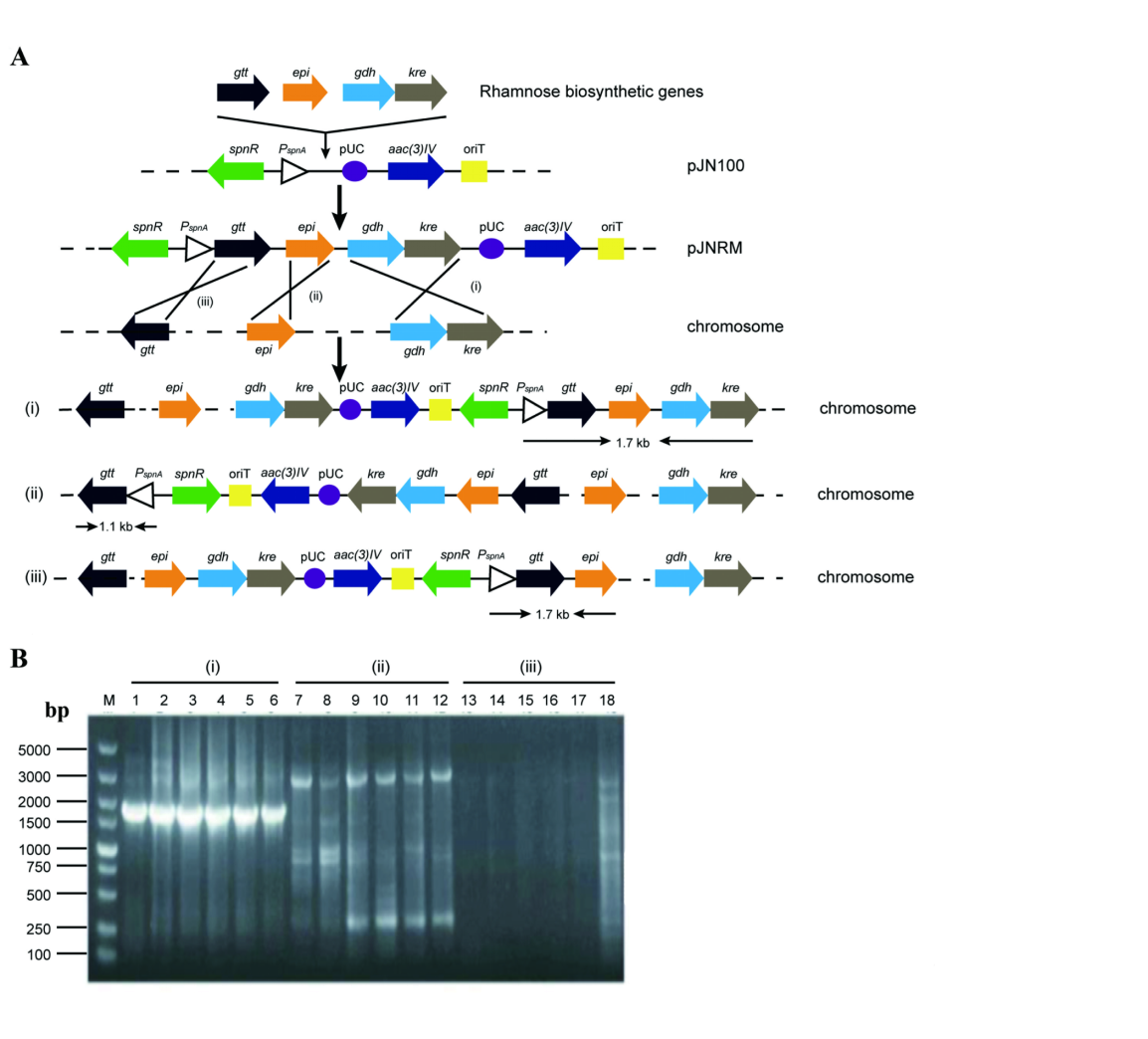


Supplementary Figure 7 | PCR analysis of integrated sites of vector pJNRM.

(A) Schematic diagram of possible ways of integration: (i) integration by 1.9 kb gdh + kre gene fragment, (ii) integration by 0.9 kb gtt gene, and (iii) integration by 0.6 kb epi gene. (B) PCR analysis of integration pattern in SPOG-RM. M, DNA Marker. Lanes 1–6, primer pair snpA-left1/gdh-in2 to verify (i) integration pattern. Lanes 7–12, primer pair snpA-left1/gtt-down2 to verify (ii) pattern. Lanes 13–18, primer pair snpA-left1/epi-down2 to verify (iii) pattern.





Supplementary Figure 8 | Construction of pKCcas9-*metK* expression vector.

1. The construction strategy of pKCcas9-*metK* expression vector. (B) Agarose gel electrophoresis analysis of *metK* (M: DNA marker. Lane 1,2: *metK*). (C) PCR identification of pKCcas9-*metK* expression vector (M: DNA marker. Lane 1: *Spe*I/*Hin*dIII double digestion).
